# Stochasticity and positive feedback enable enzyme kinetics at the membrane to sense reaction size

**DOI:** 10.1073/pnas.2103626118

**Published:** 2021-11-17

**Authors:** Albert A. Lee, William Y. C. Huang, Scott D. Hansen, Neil H. Kim, Steven Alvarez, Jay T. Groves

**Affiliations:** ^a^Department of Chemistry, University of California, Berkeley, CA 94720;; ^b^Department of Molecular and Cell Biology, University of California, Berkeley, CA 94720;; ^c^Department of Materials Science and Engineering, University of California, Berkeley, CA 94720;; ^d^Division of Molecular Biophysics and Integrated Bioimaging, Lawrence Berkeley National Laboratory, Berkeley, CA 94720

**Keywords:** enzyme kinetics, membrane, stochastic kinetics, PIP lipid, cell signaling

## Abstract

Here, we present detailed kinetic analyses of a panel of soluble lipid kinases and phosphatases, as well as Ras activating proteins, acting on their respective membrane surface substrates. The results reveal that the mean catalytic rate of such interfacial enzymes can exhibit a strong dependence on the size of the reaction system—in this case membrane area. Experimental measurements and kinetic modeling reveal how stochastic effects stemming from low molecular copy numbers of the enzymes alter reaction kinetics based on mechanistic characteristics of the enzyme, such as positive feedback. For the competitive enzymatic cycles studied here, the final product—consisting of a specific lipid composition or Ras activity state—depends on the size of the reaction system. Furthermore, we demonstrate how these reaction size dependencies can be controlled by engineering feedback mechanisms into the enzymes.

## Significance

Cellular membranes span a wide range of spatial dimensions, from the plasma membrane with a scale of microns to vesicles on the nanometer scale. The work presented here identifies a molecular mechanism, based on common features of cellular signaling enzymes, that causes the average enzymatic catalytic rate to exhibit reaction size dependency. This effect stems from stochastic variation, but the final results can be essentially deterministic. In competitive enzymatic reaction cycles, the final product can depend on the size of the reaction system. The simplicity of the mechanism suggests that size-dependent reaction rates may be widespread among signaling enzymes and thus enable reaction size to be an important factor in signal regulation at the membrane.

Enzyme kinetic reactions are commonly described in terms of deterministic rate equations. Within this type of mathematical analysis, reactant and product concentrations are treated as continuous variables, and the state of a system at any point in time is a deterministic function of the starting conditions. Even complex behaviors including bistability (1), sensitive dependence on initial conditions (e.g., chaos) (2), and spatiotemporal pattern formation (e.g., Turing instabilities) (3) can all be described with deterministic rate equations. It is computationally efficient to simulate deterministic chemical kinetics, and this method is widely used in biological sciences. For example, more than 100 papers have been published in the last 5 y analyzing Ras activation using deterministic chemical rate equations, with many of these making predictions about disease mechanisms and therapeutic approaches (4, 5). These mathematical methods, however, smooth over the fact that molecules and molecular reactions are intrinsically discrete. Moreover, the small size of cellular structures often limits physiological biochemical reactions to low molecular copy numbers, where the effects of discreteness and stochasticity become prominent.

How spatial confinement and low molecular copy numbers within cells and organelles might affect biochemical reactions has attracted significant interest over the years (6–10). However, the space of possibilities remains sparsely mapped and surprising results continue to emerge. For example, stochastic fluctuations can increase sensitivity in cellular signaling reactions (11), and they play an essential role in the bacterial chemotaxis molecular logic circuit (12). They can also induce (stochastic) bistability in systems that lack this property according to continuous kinetic rate equations (13). Recent experimental observations of a system of competing lipid kinases and phosphatases, driving interconversion between PI(4)P and PI(4,5)P_2_ in a lipid membrane, have revealed other types of macroscopic divergence from continuum kinetic predictions (14). Specifically, this system was observed to deterministically reach a PI(4)P-dominated state in large reaction systems. Under spatial confinement, however, the same system could exhibit stochastic bistability or even deterministically reach a PI(4,5)P_2_-dominated state, depending only on the size of the reaction environment. Stochastic effects led to a deterministic alteration in the average behavior, not just an increase in variation. Although stochastic kinetic modeling was able to reproduce this basic behavior, the underlying physical mechanism remains obscure. This stochastic geometry sensing mechanism also produces more elaborate pattern formations, including polarization, under different types of spatial confinement that exhibit marked similarity with living biological systems.

A distinctive feature of the competing lipid kinase–phosphatase system is that the soluble enzymes act on substrates restricted to the membrane surface. This basic reaction configuration is shared by broad classes of signal transduction enzymes in biology, including numerous protein or lipid kinases and phosphatases as well as GTPase-activating proteins (GAPs) and Guanine nucleotide exchange factors (GEFs) (15–18). For these systems, the enzyme must first contact the membrane, then find the substrate and catalyze a two-dimensional reaction at the membrane interface. This additional step offers many mechanisms for regulatory control of signaling reactions (19–21). For example, positive feedback can be easily installed on enzymes by incorporating a product binding site, which localizes the enzyme on the membrane, without the need for structural allosteric mechanisms. Other physical properties such as curvature and membrane tension can alter the enzyme activity by changing the partitioning of enzymes from the solution to the reaction surface (22, 23). Additionally, the cellular cytoskeleton and membrane topographical features can create dynamic physical barriers and confinement zones on cellular membranes (24–26). While these membrane structures are all exposed to the same cytosolic solution, the differing sizes of their effective reaction environments offer another regulatory mechanism if signaling reactions exhibit scale sensitivity.

Here, we examine a panel of soluble lipid kinases and phosphatases, as well as Ras activating proteins, acting on their respective membrane surface substrates. Using micropatterned supported lipid membranes, liposomes, and membrane-coated microbeads, we perform detailed kinetic analyses of these enzymes as a function of reaction system size. Results reveal that the mean catalytic rate of such interfacial enzymes can exhibit a strong dependence on the physical area of the membrane, which sets the copy number of enzymes within an interactive system. We find that the size dependence of the reaction rate is caused by positive feedback in the enzymatic mechanism. Furthermore, we demonstrate that size dependency can be deleted from or engineered into enzymes by deleting or adding specific lipid-binding domains. A simple analytical model, as well as more detailed stochastic kinetic simulations, reveal how size dependency of the reaction rate emerges from a coupling between positive feedback, nonequilibrium aspects of the enzymatic reaction cycle, and intrinsic stochasticity.

These basic features that lead to the size dependency of the enzymatic reaction rate are extremely common among native biological signaling enzymes. We report here that Phosphatase and Tensin Homolog (PTEN) and Phosphatidylinositol-4-Phosphate 5-Kinase (PIP5K) as well as the Ras activator Son of Sevenless (SOS) all exhibit size-dependent reaction rates. Furthermore, when coupled in a competitive enzymatic cycle, subtle differences in size sensitivity of the competing reactions can completely change the final output in a system size-dependent manner. While these experiments are done in reconstituted systems, we suggest that the underlying physical phenomenon of size-dependent enzymatic reaction rate is unavoidable in living cells.

## Results

### PTEN Exhibits Reaction System Size-Dependent Catalytic Activity.

**Fig. 1.**
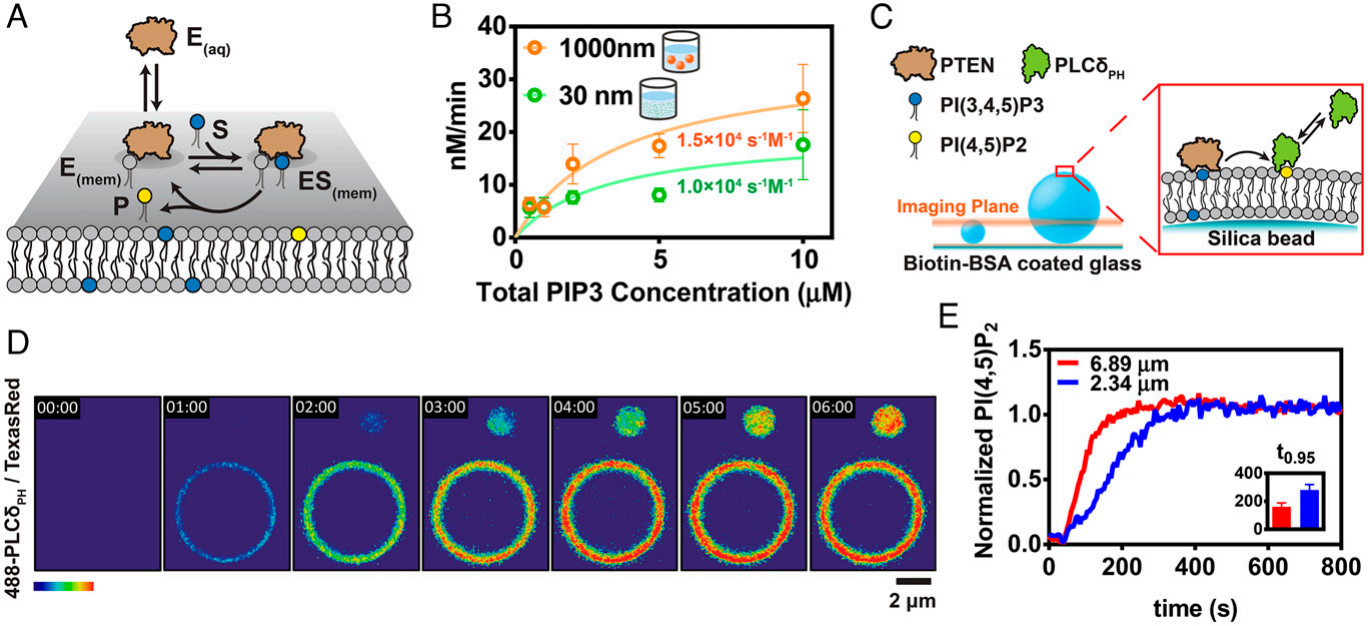
In vitro PTEN phosphatase reaction in different sizes of membrane. (*A*) Kinetic scheme of a lipid-modifying enzyme performing reaction on the membrane surface. The enzyme can dynamically bind and unbind to the membrane surface. Once bound to the membrane, the enzyme can access the substrate at the membrane and catalyze product formation following the Michaelis–Menten kinetics. (*B*) Turnover of PI(3,4,5)P_3_ to PI(4,5)P_2_ by 10 nM PTEN was measured in various total solution concentrations of PI(3,4,5)P_3_ at a fixed 2% surface concentration of PI(3,4,5)P_3_ on either 30- or 1,000-nm diameter liposomes. Fitting the data to the Michaelis–Menten equation reveals k_cat_/K_M_ values shown on the graph. (*C*) Schematic of the membrane-coated microbeads experiment setup. Microbeads coated with SLB with 96.5% of DOPC, 2% PI(3,4,5)P_3_, 1% Biotin-PE, and 0.5% TR-DHPE were tethered to glass surface functionalized with Biotin-BSA (bovine serum albumin) using neutravidin. PTEN catalyzes the conversion of PI(3,4,5)P_3_ to PI(4,5)P_2_. Production of PI(4,5)P_2_ is monitored with Alexa488-PLCδ_PH_. (*D*) Time sequence of images tracking 200 nM PTEN reaction on 6.89-μm beads and 2.34-μm beads. (*E*) Average kinetic traces of normalized PI(3,4,5)P_3_ to PI(4,5)P_2_ conversion by 200 nM PTEN plotted against time (*n* = 6). The *Inset* shows time to 95% completeness of the reactions in 6.89-μm beads and 2.34-μm beads.

Phosphatase and Tensin Homolog (PTEN) is a well-studied and important lipid-modifying enzyme (27, 28) that catalyzes the dephosphorylation of PI(3,4,5)P_3_ into PI(4,5)P_2_ and inorganic phosphate. PTEN is a soluble enzyme, which must encounter the membrane for its catalytic reaction (Fig. 1*A*) (29). PTEN contains an N-terminal PI(4,5)P_2_-binding domain (PBD), which creates a positive feedback loop in which PTEN catalyzed formation of PI(4,5)P_2_ on the membrane drives the recruitment of more PTEN to the membrane (28). Membrane localization can also lead to processivity (30, 31), in which multiple catalytic events occur during a single membrane binding dwell cycle.

We initially investigated PTEN catalytic activity on liposomes of different sizes. Liposomes consisting primarily of DOPC (1,2-dioleoyl-sn-glycero-3-phosphocholine) with 2% molar fraction of PI(3,4,5)P_3_ were prepared by extrusion through polymer filter membranes of either 30- or 1,000-nm pore size. While extrusion yields broadly dispersed liposome sizes, extrusion through 30-nm pores produces distinctly smaller liposomes than obtained from the 1,000-nm pore size (32). For the liposome assays, PTEN catalytic activity was monitored by detecting released inorganic phosphates from the reaction using a phosphate binding protein labeled with the environmentally sensitive fluorescence probe MDCC (N-[2-(1-maleimidyl)ethyl]-7-(diethylamino)coumarin-3-carboxamide), which increases fluorescence yield upon binding to inorganic phosphate (33). Kinetic traces of PTEN activity reveal that the reaction is slower in 30-nm extruded liposomes compared to 1,000-nm extruded liposomes (*SI Appendix*, Fig. S1). By fixing both the PTEN solution concentration and the PI(3,4,5)P_3_ surface concentration in the membrane, but varying the total amount of liposomes, the reaction velocity was mapped to overall substrate concentration (Fig. 1*B*). The apparent enzyme catalytic efficiency can be obtained by fitting the resultant reaction velocity traces to a Michealis–Menten kinetic analysis (see *Materials and Methods*). The catalytic efficiency (k_cat_/K_M_) of PTEN is increased by 50% when reacting on 1,000-nm pore extruded liposomes compared with liposomes obtained from 30-nm pore extrusion. The same size-dependent effect was also evident on membrane-coated microbeads (34), where PI(4,5)P_2_ production was monitored by imaging the binding of the fluorescently labeled PH domain of phospholipase C δ (PLCδ) to PI(4,5)P_2_ using confocal microscopy, normalized by the fluorescence from a lipid-linked Texas Red fluorophore (Texas Red 1,2-Dihexadecanoyl-sn-Glycero-3-Phosphoethanolamine) present in the membrane at a fixed density (0.5%) (Fig. 1*C*). Under the experimental conditions used both here on microbeads and in the supported membrane corral arrays described later in this section, generally less than 0.1% of PI(4,5)P_2_ lipids are bound by the fluorescent probe at any given time (14). After PTEN is added, PI(4,5)P_2_ is produced at faster rates in membrane-coated microbeads with a larger diameter (Fig. 1 *D* and *E* and Movie S1). The time to 95% completeness of reaction is 80% longer in 2.34-μm beads compared to 6.89-μm beads.

**Fig. 2.**
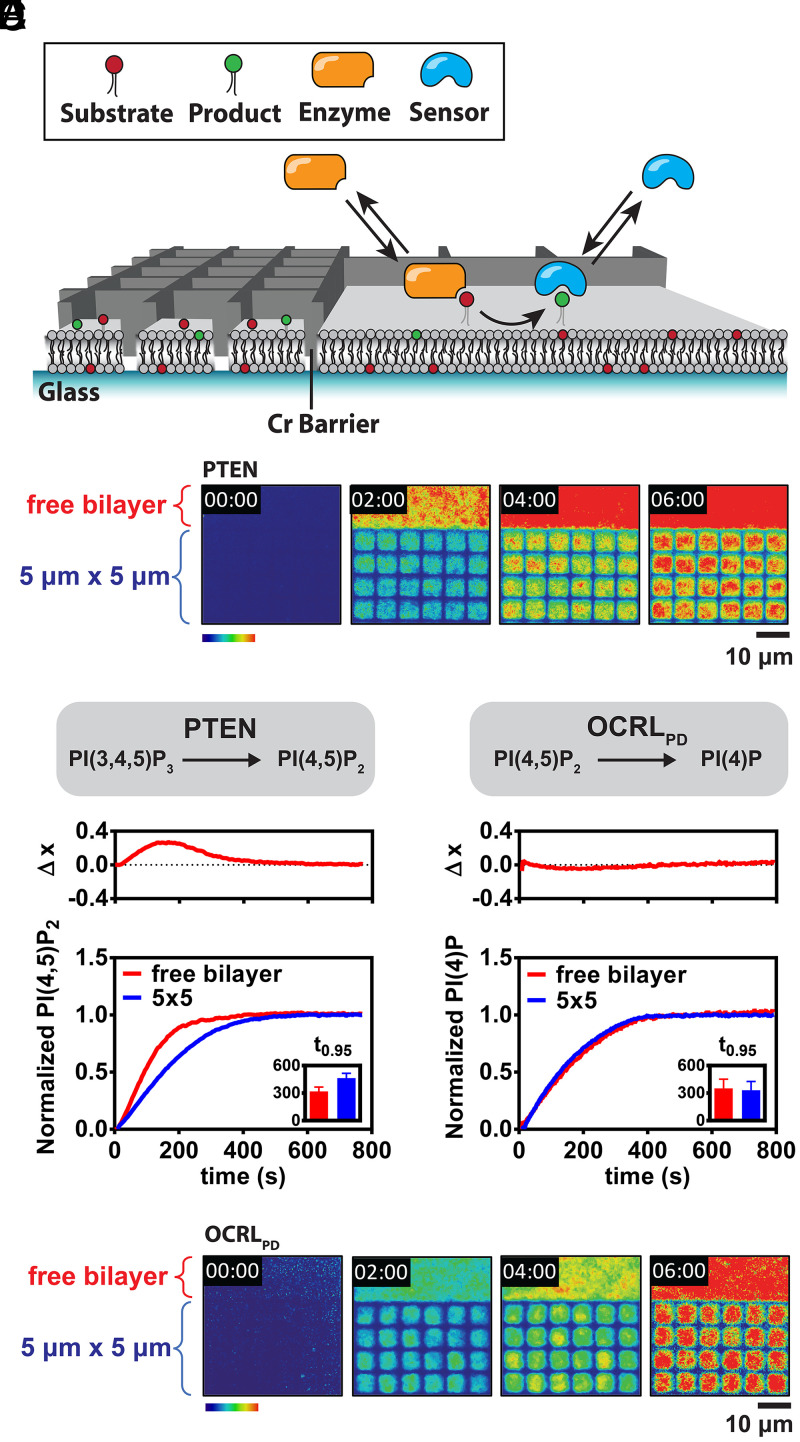
Kinetics of enzymes on confined planar lipid bilayer. (*A*) Scheme of the SLB experimental setup. Nanofabricated chromium barriers partition a supported bilayer into micrometer-scale corrals, each containing identical composition. When the enzyme converts substrates to products at the membrane, the products can be monitored with a fluorescently labeled lipid binding domain that binds to the product. (*B*) Time sequence of images of 100 nM PTEN reaction on membrane containing 2% PI(3,4,5)P_3_ monitored by 20 nM Alexa488-PLCδ_PH_. Reactions in 5 × 5 μm membrane corrals were imaged alongside with reaction in free bilayer in the same experiment. (*C*) Dephosphorylation reaction of PI(3,4,5)P_3_ to PI(4,5)P_2_ by 100 nM PTEN in 5 × 5 μm of membrane corrals and free bilayer plotted together. *Top* graph: the difference between the reaction trajectory in the free bilayer and 5 × 5 μm membrane corrals, Δx, at each time point. The *Inset* shows time to 95% completeness of the reactions in free bilayer and 5 × 5 μm membrane corrals. (*D*) Time sequence of images of 50 nM OCRL_PD_ reaction in membrane containing 4% PI(4,5)P_2_ monitored by 20 nM Cy3-DrrA. (*E*) Dephosphorylation reaction of PI(4,5)P_2_ to PI(4)P by 50 nM OCRL_PD_ in 5 × 5 μm membrane corrals and free bilayer.

Changing the diameter of liposomes or microbeads not only changes membrane surface area but also curvature. Since membrane curvature can significantly change the reaction rate of some enzymes (21), we implemented the PTEN activity assays in a planar micropatterned supported lipid bilayer (SLB) format (Fig. 2*A*) (35). Grids of chromium metal lines, prefabricated onto glass coverslips, create barriers to support membrane formation and effectively confine the membrane into two-dimensional corrals with micrometer-scale dimensions (31, 36). Lipids and membrane-associated proteins diffuse freely within each confined corral but cannot cross the barriers. However, all corrals are in contact with the same bulk solution, and the low vertical height of the metal lines (∼9 nm in these experiments) has essentially no effect on the diffusion and flow of molecules in the bulk solution phase. The SLB experimental system provides superior subsecond time resolution and control of reaction size homogeneity compared to the liposome and bead assays (14). Moreover, the system is completely planar, leaving the surface area and shape to be the only geometrical variables.

The catalytic activity of PTEN was observed in the unrestricted free lipid bilayer, with a scale on the order of millimeters, and in 5 × 5 μm corralled membrane arrays. Confinement grids were patterned side by side with the unrestricted regions, enabling simultaneous monitoring in both regions under identical reaction conditions (Fig. 2*B* and *SI Appendix*, Fig. S2*A* and Movie S2). PTEN and the lipid sensor were introduced into the system from the solution flowed into the flow cell. All regions of the supported membrane are in contact with the exact same solution above. Under these conditions, restricting the membrane surface reaction size from the free lipid bilayer to 5 × 5 μm corrals significantly slows down the mean reaction rate. This is evident in the total internal reflection fluorescence (TIRF) intensity plots—mapping PI(4,5)P_2_ membrane concentration—illustrated in Fig. 2*B*. At 4 min into the reaction, the bulk membrane area is nearly completely converted to PI(4,5)P_2_ while each of the corralled membrane regions lags significantly. This kinetic experiment is quantified in Fig. 2*C* where the mean normalized PI(4,5)P_2_ density is plotted versus time for corralled and free membrane regions (replicates shown in *SI Appendix*, Fig. S2*A*). The maximum difference in normalized reaction progress (Δx) across the reaction period can reach more than 0.2. Since all membrane regions in this experiment are entirely flat, membrane curvature is ruled out as a cause of the differential enzyme efficiency. Membrane surface area alone is sufficient to cause the difference in reaction speed.

As will be discussed in further detail in the last two sections, this size-dependent enzymatic reaction speed is fundamentally the result of stochastic effects in enzyme copy number on the membrane surface. However, it is important to note that observed reaction rates do not vary substantially from corral to corral in the 5 × 5 μm array. Each corral confined reaction is consistently slower than the unrestricted membrane (*SI Appendix*, Fig. S2*A*).

Size-dependent reaction speed is a property of the enzyme and is not universal. Similar experiments on another lipid phosphatase, phosphatidylinositol 5'-phosphatase domain of Lowe Oculocerebrorenal Syndrome Protein (OCRL_PD_), do not exhibit reaction size–dependent effects. Kinetic traces of OCRL_PD_ catalyzed reactions on the bulk membrane and in 5 × 5 μm corral arrays are essentially identical, exhibiting Δx values below 0.05 throughout the reaction (Fig. 2 *D* and *E* and *SI Appendix*, Fig. S2*B* and Movie S3).

### Positive Feedback Enables Size-Dependent Catalytic Activity.

**Fig. 3.**
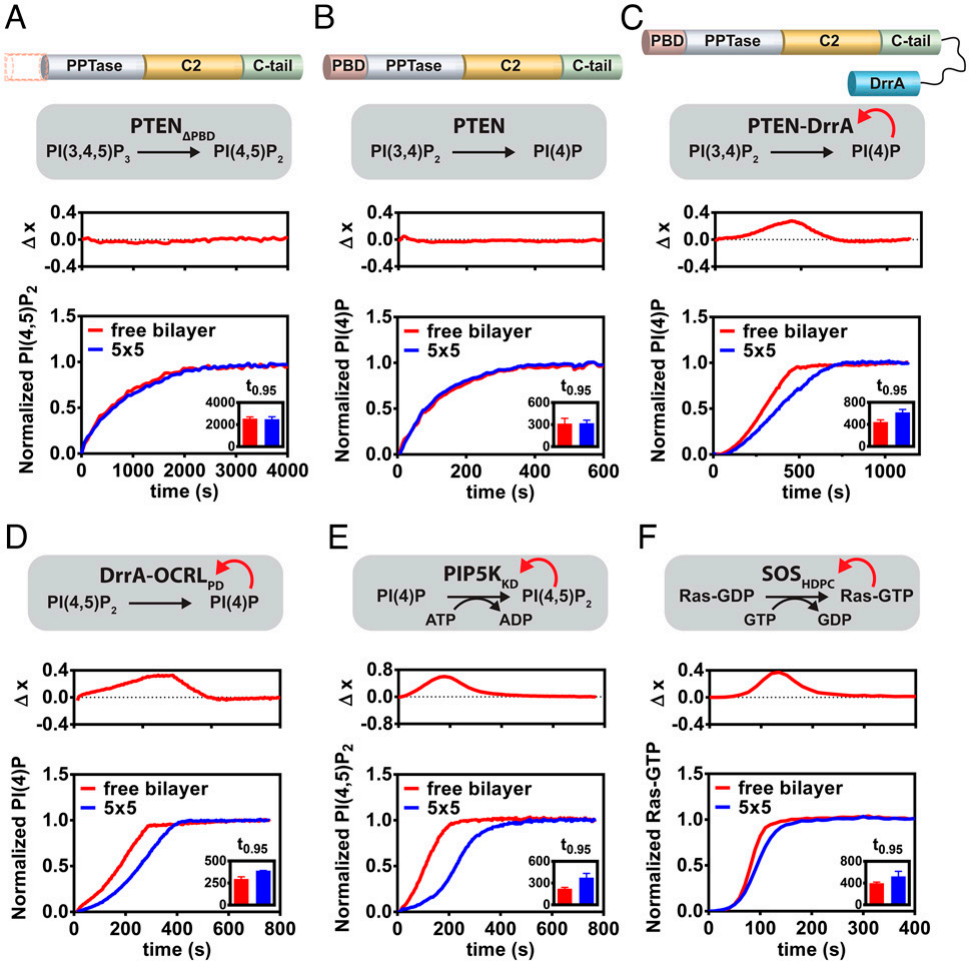
Biochemically engineering size-dependent reaction speed. (*A*) Dephosphorylation reaction of PI(3,4,5)P_3_ to PI(4,5)P_2_ by 23 μM PTEN_ΔPBD_ in 5 × 5 μm membrane corrals and free bilayer. PBD: PIP2-binding domain; PPTase: Phosphatase domain; C-tail: C-terminal tail. (*B*) Dephosphorylation reaction of PI(3,4)P_2_ to PI(4)P by 3 μM PTEN in 5 × 5 μm membrane corrals and free bilayer. (*C*) Dephosphorylation reaction of PI(3,4)P_2_ to PI(4)P by 170 nM PTEN-DrrA in 5 × 5 μm membrane corrals and free bilayer. (*D*) Dephosphorylation reaction of PI(4,5)P_2_ to PI(4)P by 100 pM DrrA-OCRL_PD_ in 5 × 5 μm membrane corrals and free bilayer. (*E*) Phosphorylation reaction of PI(4)P to PI(4,5)P_2_ by 2 nM PIP5K_KD_ in 5 × 5 μm membrane corrals and free bilayer plotted together. (*F*) Nucleotide exchange reaction of Ras-GDP to Ras-GTP catalyzed by 20 nM SOS_HDPC_ in 5 × 5 μm membrane corrals and free bilayer plotted together. The reaction is monitored by binding of a fluorescently labeled Ras-binding domain.

We characterized the mechanistic origin of PTEN reaction size sensitivity by first removing its positive feedback. In the PTEN domain structure, the PBD domain is followed by phosphatase, C2, and C-terminal domains (27). We truncated the PBD domain to construct the PTEN_ΔPBD_ variant, which lacks the PI(4,5)P_2_ membrane binding–mediated positive feedback loop (Fig. 3*A*). The activity of PTEN_ΔPBD_ is significantly compromised and no activity was observable on 2% PI(3,4,5)P_3_ lipid membranes (*SI Appendix*, Fig. S3). Increasing the overall membrane negative charge by adding 5% PS, in addition to the 2% PI(3,4,5)P_3_, facilitated the reaction and revealed that PTEN_ΔPBD_ does not exhibit size dependency (Fig. 3*A* and *SI Appendix*, Fig. S2*C* and Movie S4). Control experiments including PS with full-length PTEN exhibit the same size dependency observed on 2% PI(3,4,5)P_3_ membranes, confirming that PS is not responsible for inhibiting reaction size sensitivity (*SI Appendix*, Fig. S4). The PTEN PBD domain is essential for its reaction size–dependent catalytic activity.

The apparent primary function of the PTEN PBD domain is to mediate membrane recruitment by binding PI(4,5)P_2_, providing a positive feedback loop. However, it remains unclear if the inability of PTEN_ΔPBD_ to exhibit size-dependent activity is solely caused by loss of positive feedback or other unknown functions of the PBD. To investigate this, we constructed a reaction system with native PTEN but in which the PI(4,5)P_2_ positive feedback loop is eliminated. PTEN phosphatase activity is promiscuous, and it readily catalyzes 3'-dephosphorylation of not only PI(3,4,5)P_3_ but also other phosphatidylinositols containing 3'-phosphate, such as PI(3,4)P_2_ to PI(4)P(37). PBD binding, however, is quite specific and only PI(4,5)P_2_ strongly activates PTEN while other phosphatidylinositols, including PI(3,4)P_2_, either do not activate or only weekly activate PTEN (37, 38). Therefore, without any PI(4,5)P_2_-mediated activation, PTEN catalyzed 3'-dephosphorylation of PI(3,4)P_2_ to PI(4)P cannot exhibit strong positive feedback. As anticipated, kinetic analysis of PTEN catalyzed PI(3,4)P_2_ to PI(4)P reactions in the bulk membrane and in 5 × 5 μm corral arrays also do not exhibit any detectable size-dependent catalytic activity (Fig. 3*B* and *SI Appendix*, Fig. S2*D* and Movie S5).

By engineering a PI(4)P-binding domain into PTEN, we constructed a variant with positive feedback in the PI(3,4)P_2_ to PI(4)P reaction. DrrA is a GEF of Rab1 that contains a PI(4)P-binding domain (DrrA 544 to 647) (39). We refer to this fragment as DrrA hereafter. Kinetic traces from the PTEN–DrrA reaction on 2% PI(3,4)P_2_ membrane follow a strongly sigmoidal shape, indicating the reaction has positive feedback. Starkly contrasting PTEN, PTEN–DrrA shows strong reaction size–dependent catalytic activity in the 3'-dephosphorylation of PI(3,4)P_2_ (Fig. 3*C* and *SI Appendix*, Fig. S2*E* and Movie S6). Using a similar strategy, the OCRL_PD_ catalyzed PI(4,5)P_2_ to PI(4)P dephosphorylation reaction, which intrinsically lacks feedback, can be augmented with positive feedback by fusing OCRL_PD_ with a DrrA domain. Kinetic traces of DrrA–OCRL_PD_ show both positive feedback and size-dependent reaction speed (Fig. 3*D* and *SI Appendix*, Fig. S2*F* and Movie S7). Overall, these data illuminate a clear and causal relationship between membrane binding–mediated positive feedback and reaction size dependency of catalytic activity.

Across the wide variety of chemical reactions catalyzed by interfacial enzymes, positive feedback through product binding is a common feature among many of them. In addition to lipid phosphatases such as PTEN, lipid kinases such as PIP5K, and GEFs such as SOS have all been reported to natively possess such a mechanism (14, 40). We therefore posited that these enzymes all could exhibit reaction size dependency in their catalytic activity and tested this with the kinase domain of PIP5K (PIP5K_KD_) and the catalytic N-terminal fragment of SOS (SOS_HDPC_). PIP5K_KD_ catalyzes PI(4)P to PI(4,5)P_2_ reaction at the expense of an ATP and separately binds PI(4,5)P_2_. SOS_HDPC_ catalyzes nucleotide exchange, converting Ras-GDP to Ras-GTP and can bind Ras-GTP with a stronger affinity at an allosteric site (36). Both PIP5K and SOS showed size-dependent catalytic activity (Fig. 3 *E* and *F* and *SI Appendix*, Fig. S2 *G* and *H* and Movies S8 and S9). Notably, while the catalytic domain of SOS, SOS_cat_, contains the allosteric Ras-GTP (product) binding site and showed clear positive feedback in its catalytic activity, it is not size sensitive under the conditions in our experiment (*SI Appendix*, Fig. S5 and Movie S10). As will be clarified in the last section, this can be attributed to the fact that SOScat is distinctively less processive than either SOSHDPC or native full-length SOS (36, 41, 42). While strong processivity is neither a requirement nor sufficient for reaction size sensitivity, it is an amplifier of these effects.

### Competitive Enzymatic Cycles Amplify Effects of Reaction Size Dependency.

Native forms of all of the enzymes studied here operate in competitive reaction cycles under physiological conditions. Kinases are opposed by phosphatases, Ras GEFs are opposed by GAPs, and this is a common theme across many biological signaling systems. In such competitive reactions, small differences in reaction rate can determine what the final outcome is, and this can amplify the consequences of even small reaction size dependencies among the competing enzymes. As an example of this, we here study the competitive reaction between PIP5K and OCRL. This system drives interconversion between PI(4)P and PI(4,5)P_2_ and is one of several similar competitive lipid kinase–phosphatase systems we have recently found to exhibit complex reaction size sensitivity and pattern forming tendencies (14).

**Fig. 4.**
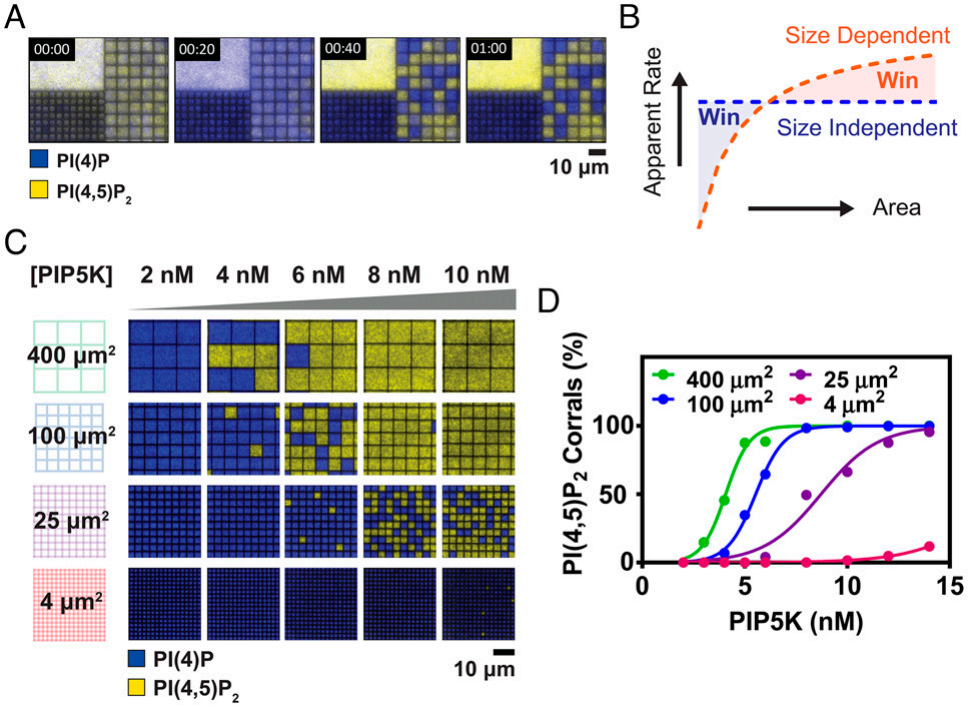
Size-dependent reaction speed controls the reaction outcome in a competition reaction. (*A*) Time sequence of the competition reaction of 10 nM PIP5K and 700 nM OCRL monitored by 20 nM Alexa488-PLCδ_PH_ and 20 nM Cy3-DrrA in 2 × 2 μm corrals and 5 × 5 μm corrals, as well as the unrestricted free bilayer. (*B*) Size-dependent reaction speed in a competition reaction can lead to a change of reaction outcome based on size. (*C*) Images of the final steady-state outcome for a series of 4% PI(4)P membrane with surface areas ranging from 400 to 4 μm^2^ when exposed to various concentrations of PIP5K and a fixed 1 μM OCRL. (*D*) The probability of the PI(4,5)P_2_ enriched reaction outcome for a series of 4% PI(4)P membrane with different dimensions at 1 μM OCRL and various concentrations of PIP5K. The data are fitted with a general sigmoidal equation.

A time sequence of images following a reaction trajectory for the PIP5K:OCRL system on SLB corral arrays of various sizes is illustrated in Fig. 4*A* (Movie S11). For these experiments, the supported membrane has an initial composition of 2% PI(4)P and 2% PI(4,5)P_2_ (in a DOPC background), and lipid sensors for PI(4)P (DrrA), in blue, and PI(4,5)P_2_ (PLCδ) in yellow track the composition over time, by TIRF imaging. The reaction is initiated by injecting a solution of both enzymes, ATP, and lipid sensors into the imaging flow cell. As can be seen in the image sequence, the larger area of the membrane is smoothly driven to a PI(4,5)P_2_-dominated state, indicating that the average balance between kinase and phosphatase in this particular experiment favors the kinase. However, under the identical enzyme mixture in the solution, the system exhibits bistability in 5 × 5 μm corral arrays and is uniformly driven to a PI(4)P-dominated state in 2 × 2 μm corral arrays. In this case, the net reaction outcome—a PI(4)P- or PI(4,5)P_2_-dominated state—depends on the size of the membrane reaction system. This effect can drive reaction outcome with near certainty; note that there are no visible 2 × 2 μm corrals ending in the PI(4,5)P_2_-dominated state even though this is the kinetically favored state in the bulk average.

The complete inversion in the outcome of the PIP5K:OCRL system, as a function of reaction size, is achieved based on differences in the size dependency of the individual enzymatic reactions. In this case, PIP5K has positive feedback and exhibits size-dependent reaction rates whereas OCRL does not. The effect of reaction size on the balance between these two reactions is illustrated schematically in Fig. 4*B*. For a given enzyme concentration in solution, the reaction rate for PIP5K increases with reaction size while that of OCRL is constant. As such, it is possible to achieve a situation in which positive feedback in PIP5K provides it with a kinetic advantage in large systems, while OCRL can still dominate in sufficiently small systems. We note that in our previous study of a similar system with PIP5K, many of the experiments utilized variants of OCRL with engineered positive feedback (14). In those experiments, both enzymes exhibit positive feedback and size sensitivity. The particular balance between size sensitivity of the competing enzymes led to exactly the opposite size preference seen here: PIP5K selectively dominated in small corrals. These contrasting results underscore how controllable the size dependency of enzymatic reaction rates can be.

Activation of membrane signaling in physiological systems often involves increasing the activity of a kinase to overcome the suppressing activity of phosphatases. Effects of reaction size confinement on this balance for the PIP5K:OCRL system are illustrated through a set of PIP5K titration experiments shown in Fig. 4*C*. The competitive reaction is run on a series of membrane corral arrays, spanning a factor of 100 in surface area (2 × 2 μm to 20 × 20 μm), at fixed OCRL concentration (1 μM) and a series of PIP5K concentrations ranging from 2 to 10 nM. Although in all cases, the competitive reaction exhibits two well-defined possible outcome states, the PIP5K concentration at which switching between these states occurs exhibits a sharp dependence on reaction size (Fig. 4*D*). The size range we tested here resembles the length scale of larger geometrical features in cellular systems, such as filopodia, lamellipodia, and polarization in the plasma membrane. The concentration range of PIP5K also falls within physiological expression levels (43).

The competitive reaction between Ras activation by SOS and deactivation by the p120 Ras GAP exhibits a similar size dependency of reaction outcome (*SI Appendix*, Fig. S6). This effect is observed for SOS_HDPC_ but not SOS_cat_ (*SI Appendix*, Fig. S7) and follows consistently with our observation that SOS_HDPC_ exhibits substantially greater size-dependent activity than SOS_cat_. Both of these SOS constructs have positive feedback, but their difference lies in the degree of processivity. The lipid-binding properties of SOS_HDPC_ enable it to linger at the membrane for longer dwell times than SOS_cat_ in these experiments. As such, stochastic variation in enzymatic reaction rate resulting from enzyme binding and desorbing from the membrane is amplified for SOS_HDPC_ relative to SOS_cat_, and these stochastic fluctuations are key to the strength of reaction size dependency. Note that SOS_cat_ and SOS_HDPC_ are truncated forms of SOS and that the native full-length SOS protein is extremely processive (36, 41, 42).

### Size Dependency of Reaction Rate Arises from a Stochastic Mechanism.

**Fig. 5.**
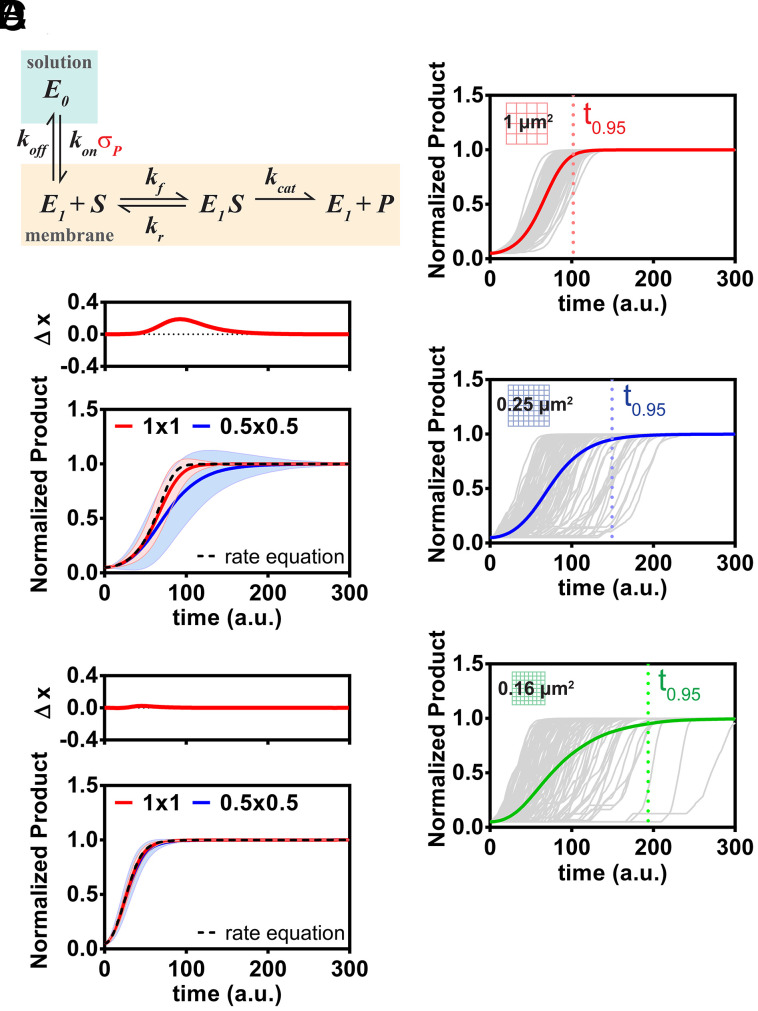
Size-dependent reaction speed modulated by reaction discreteness and enzyme processivity. (*A*) Kinetic scheme for stochastic kinetic modeling. The interfacial enzyme binds to a membrane from the solution and catalyzes a surface Michaelis–Menten reaction at the membrane. Positive feedback is included by having the enzyme on rate dependent on σ_P_ (density of product on the membrane). (*B*) Kinetic traces from 1,000 stochastic simulations plotted with their mean (colored lines) using the reaction mechanism described in *A*. (*Top*) Simulation in 1-μm^2^ membrane. (*Middle*) Simulation in 0.25-μm^2^ membrane. (*Bottom*) Simulation in 0.16-μm^2^ membrane. t_0.95_ marks the time for the average to reach 95% completeness of reaction. (*C*) Average of kinetic traces from stochastic simulations in 1 × 1 μm (1 μm^2^) membrane and 0.5 × 0.5 μm (0.25 μm^2^) membrane plotted together. Simulation with deterministic rate equation was plotted in dashed line. The shaded area shows the SD. The *Top* graph shows the difference between the reaction trajectory in 1- and 0.25-μm^2^ arrays, Δx, at each time point. The difference of normalized product at max Δx is significant at *P* value <0.01. (*D*) Simulation with no positive feedback (on rate independent of σ_p_).

We investigate the underlying mechanism of reaction size dependence of catalytic activity with stochastic kinetic modeling of the basic Michaelis–Menten enzymatic process. The reaction scheme for the interfacial enzymes considered here is depicted in Fig. 5*A*. The enzyme in solution (*E*_0_) is recruited to and desorbs from a membrane-bound state (*E*_1_) via overall kinetic rate parameters (*k*_on_ and *k*_off_), which are not necessarily constants since they may depend on membrane composition (e.g., concentration of the enzymatic product). On the membrane, the enzyme interacts with the substrate (*S*), forming an enzyme–substrate complex (*E*_1_:*S*) with overall kinetic rates (*k*_f_ and *k*_r_), from which the product is formed with a catalytic rate constant (*k*_cat_). We perform stochastic kinetic modeling of this reaction scheme using a Gillespie algorithm (44), describing the state vector for the system in terms of discrete copy numbers of each species on the membrane (*E*_1_, *S*, *E*_1_:*S*, *P*). The concentration of the solution species, *E*_0_, is fixed, reflecting the experimental condition where there is a large reservoir of enzymes in solution. Transitions between states are described with transition probabilities, corresponding with each of the kinetic rates, some of which are functions of the state of the system (full detail in *Materials and Methods*). This modeling is spatially homogeneous (matching experimental conditions), and the system size in spatial dimensions maps to different overall molecular copy numbers in the simulations.

Stochastic kinetic modeling readily reproduces the experimental observation of reaction size–dependent catalytic activity, while deterministic rate equations fail to predict such effects. Sets of reaction trajectories for the same enzymatic system in differently sized membrane corral arrays are shown in Fig. 5*B*. As expected, stochastic variation clearly becomes more pronounced in the smaller corrals. More importantly, the mean catalytic activity also differs. Mean reaction trajectories from these simulations on 1 and 0.25 μm^2^ arrays are plotted in Fig. 5*C* in the same format used for the presentation of experimental data in Fig. 2, illustrating the substantial agreement between modeling and experiment results (reference *SI Appendix* for discussion). If membrane binding of the enzyme is decoupled from product density, effectively removing the positive feedback, size dependency of the reaction rate is lost (Fig. 5*D* and *SI Appendix*, Fig. S8).

**Fig. 6.**
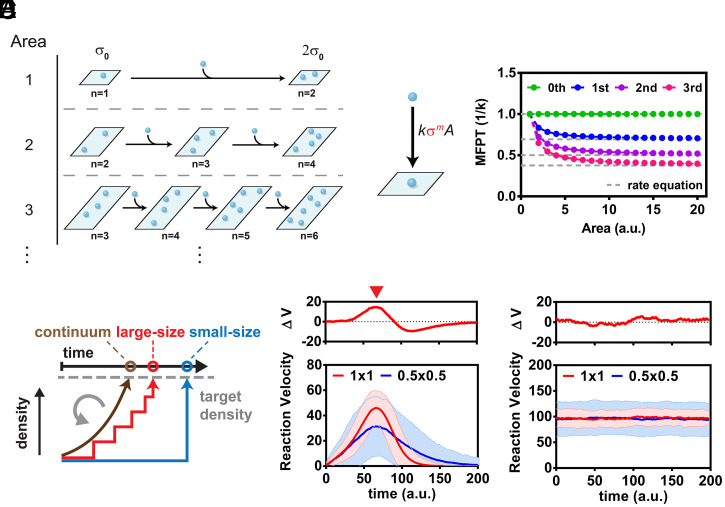
Size-dependent reaction speed based on positive feedback through recruitment. (*A*) A kinetic model of a surface binding process. Surfaces with different areas start with the same density σ_0_ and can bind molecules with a defined kinetic rate to evolve to density state 2σ_0_. (*B*, *Left*) The kinetic scheme for direct positive feedback binding. *k*σ*^m^A* is the binding rate. *m* is the order of positive feedback. In the case of *m* = 0, there is constant membrane binding with no positive feedback. (*Right*) The MFPT for different surface areas to reach σ = 2 with direct positive feedback binding kinetics. (*C*) Membrane size alters the continuity of positive feedback. Discontinuity becomes more prominent in smaller system sizes. Deviation from continuous positive feedback leads to a weaker positive feedback and slower overall reaction. (*D*) Fig. 5*C* plotted in reaction velocity versus time. The shaded area shows the SD. The *Top* graph shows the reaction velocity difference (ΔV) between 1- and 0.25-μm^2^ arrays. The red arrow indicates the time point where ΔV is at maximum. (*E*) Stochastic simulations at a fixed substrate and product densities plotted in reaction velocity versus time. The condition corresponds to the membrane composition at the red arrow in *D*.

To conceptually illustrate the underlying physical mechanism of size dependency in reaction rate, we construct a highly simplified stochastic system that still exhibits the basic effect. In this example, consider a molecule that binds to a surface in a one-way process with a kinetic rate that depends on the surface concentration of already bound molecules (positive feedback) (Fig. 6*A*). We can examine the overall reaction rate by looking at the mean first passage time (MFPT) for the system to double the density of adsorbed molecules (σ). Fig. 6*A* depicts the density doubling process (copy number n molecules goes to 2n) for several different sized systems, starting from n = 1, 2, or 3 adsorbed molecules, at equivalent starting surface density. The number of individual molecular binding events required for density doubling goes as *n*, and the probability distribution for doubling time, τ_D_, is given by successive convolution of the individual transition time distributions for each of the *n* transitions: $p(\tau_{D})=p_{1}(\tau_{1})⊗p_{2}(\tau_{2})⊗p_{3}(\tau_{3})\cdots ⊗p_{n}(\tau_{n})$. For this one-way adsorption process, the MFPT for doubling is simply the average doubling time, $〈\tau_{D}〉$, and since this is a Markov process, $〈\tau_{D}〉=\sum_{i=1}^{n}〈\tau_{i}〉$.

For the case of simple binding, with no feedback, the overall rate of binding to a surface with area, A, is independent of the number of already adsorbed molecules and given by kA. With this constant rate of binding, the delay time between each of the individual binding events follows an identical Poisson interval distribution, $p(\tau )=kAe^{-kA\tau }$ and $p(\tau_{D})$ is the corresponding gamma distribution: $p(\tau_{D})={\left(kA\right)}^{\left(n+1\right)}\tau_{D}{}^{n}e^{-kA\tau }/n!$. In this case with zero-order feedback, the MFPT for doubling is independent of system size and identical to the value calculated from a continuum approach with deterministic rate equations (Fig. 6*B*; also see *Materials and Methods*).

When there is positive feedback (of order m) affecting the adsorption process, the MFPT for density doubling is calculated as above, except now the intermediate transitions no longer occur with an identical rate. For a system starting with n molecules, the $i^{th}$ transition has rate $k{\text{σ}}^{m}A$, where σ=(n+i−1)/A is the momentary density of adsorbed molecules while waiting for the $i^{th}$ transition event. The rate of each successive step now depends on σ and correspondingly increases, reflecting the positive feedback as a function of already adsorbed molecules. Plots of doubling MFPT versus system size for feedback of order m = 1, 2, and 3 are shown in Fig. 6*B*. With the positive feedback, a system size dependence of the overall reaction rate is evident with the reactions going more slowly in smaller systems. At larger system sizes, the stochastic analysis converges on the same result (dashed lines) obtained from continuum deterministic rate equations.

Fundamentally, stochastic effects originating from the discrete binding of molecules to the surface reduce the efficiency of the positive feedback. In the extreme case of beginning with a single molecule, the MFPT for doubling essentially never experiences any effects of feedback since the process is finished with the first transition. As systems get progressively larger, and more individual steps are taken throughout the reaction trajectory to achieve the same density doubling, each successive step occurs faster as the system is able to respond to the now gradually increasing density (Fig. 6*C*). Effects of feedback are maximized in large systems, where the surface density of adsorbed molecules essentially varies continuously.

### Size Dependency of Reaction Rate Is a Nonequilibrium Effect.

In addition to the stochastic element, the mechanism of reaction size dependency is also intrinsically rooted in the fact that the system is changing. This is clearly demonstrated by examining reaction velocities under steady-state conditions (e.g., as might be done in some classic Michaelis–Menten analyses). Fig. 6*D* illustrates a plot of mean reaction velocity versus reaction progress for the 1- and 0.25-μm^2^ corral arrays for the system computationally analyzed in Fig. 5 *B* and *C*. Marked on the plot is the system composition (substrate and product densities) at which the maximum difference in mean reaction velocity between the two corral sizes was observed. Results from stochastic simulations on the two corral sizes at this composition but now under steady-state conditions (with substrate and product densities fixed) are plotted in Fig. 6*E*. As expected, reaction velocity variation is substantially larger in the smaller corrals. However, under these steady-state conditions, there is no longer any size dependency of the mean reaction rate.

The reaction size–dependency effect stems from the enzyme–membrane binding reaction being out of equilibrium and the way in which this binding reaction stochastically follows the changing membrane composition. This can be shown by running stochastic simulations for the system depicted in Fig. 5*C* in which the positive feedback is preserved, but the individual kinetic rate constants for membrane binding are allowed to be very fast compared to the catalytic rate (increasing both *k*_on_ and *k*_off_ or reducing *k*_f_ and *k*_cat_). In these situations, the enzyme–membrane binding reaction is always near equilibrium (or quasi steady state), and the reaction size dependency correspondingly vanishes (*SI Appendix*, Fig. S9). In sufficiently small systems, where individual catalytic steps can appreciably change the system product density, nonequilibrium behavior is essentially assured since the enzyme–membrane binding reaction cannot synchronously follow the stochastic steps of the catalytic reaction. However, we also observe reaction size sensitivity in systems with relatively high substrate density (e.g., Fig. 5*C*). In these cases, the enzymes are significantly processive, driving more than 100 catalytic cycles per binding event at the early stages of the reaction. This dramatically amplifies stochastic variation in the overall reaction velocity and correspondingly amplifies size sensitivity.

We note that positive feedback and a nonequilibrium reaction cycle are the necessary and sufficient conditions for reaction size sensitivity. Enzymatic processivity is an amplifier of these effects but is not required. Reaction size sensitivity can be readily detected without any processivity at sufficiently low total substrate density (*SI Appendix*, Fig. S10).

## Discussion

The impacts of size and geometry of cellular structures on intracellular biochemical reactions and signaling processes have previously been considered in a variety of different contexts. For example, cell shape has been shown to direct stronger cyclic adenosine monophosphate (cAMP) signaling in the dendrites of neuronal cells through the interplay between reaction diffusion and changes in surface to volume ratios (45). In an analogous mechanism, mitogen-activated protein kinase (MAPK) phosphorylation level upon EGF stimulation can be enhanced in elliptic cells compared to circular cells (46). In these examples, where the reaction occurs at the membrane but the effector diffuses through solution, effective changes in local surface area to volume ratios caused by membrane bending and cell shape can establish zones of higher reactivity or depletion of the effectors and consequently alter local or global reaction outcome (6, 22, 45, 46). Size sensing behavior has also been reported in the depolymerization of microtubules (47) and actin filaments (48). In these cases, the size-dependent effects originate from a lower dimensional version of the surface area to volume ratio—the length to end-point ratio. Elongating filament length increases the number of available binding sites per filament and recruits more enzymes. Processive movement of the enzyme toward the end of the filament then leads to concentrated activity at the end of the filament that is proportionate with its length. The size sensitivity in mean catalytic rate that we report here, however, is quite distinct from these other processes. It is not dependent on an interdimensional ratio, such as surface area to volume, nor are there any requirements on diffusion or enzymatic processivity. Also, unlike the examples mentioned above, and the beautiful spiraling Turing patterns exhibited by the Min system (49), the size sensitivity we describe is not predictable by continuum mathematical descriptions of reaction kinetics: this size sensitivity is intrinsically stochastic.

The patterned supported membrane experimental platform provides a unique way to isolate the effects of system size from other geometrical features, such as membrane curvature. For the lipid kinase–phophatase reactions studied here, this resolving capacity confirms that it is size, not curvature, that led to the observed differential kinetic rates. However, membrane curvature is a major aspect of physiological membrane systems, and there is significant interest in curvature driven effects. Studies on the regulation of lipase and phospholipase activity by curvature are notable examples. Classical as well as modern research performed on this topic mainly utilizes liposomes of different sizes to represent different curvatures, leading to discoveries of diverse curvature sensing mechanisms (22, 23). Depending on feedback characteristics of the enzymes (50), such experimental observations may also be influenced by size-dependent reaction effects as described here. Planar supported membrane microarrays could be useful in control experiments to distinguish these mechanistic details.

We have demonstrated that even the extremely minimal system consisting of an ensemble of identical soluble enzymes acting on a membrane substrate can exhibit a reaction size–dependent mean catalytic rate. For a pair of such enzymes in a competitive reaction, this effect can lead to complete reaction inversion, in which the final product depends on the system size. Although these effects arise through a stochastic mechanism, the results are not random and can be achieved with almost complete certainty. Reaction size dependency of an interfacial enzymatic reaction emerges when two conditions are met: 1) the enzyme exhibits feedback, and 2) the intermediate binding interaction between enzyme and membrane is not well equilibrated with the changing membrane composition. Feedback is a genetically encodable (and engineerable) property of the enzyme while the nonequilibrium characteristic is a property of the reaction system. These requirements are so basic, and simply met, that we suggest it is unavoidable that they occur within cells and possibly govern some biological functions. In addition to the enzymatic reactions studied here, many important signaling events that involve the activation of membrane substrate by soluble enzymes, such as activation of Cdc42 (51, 52), RhoA (53), Rab-5 (54), Arf-1, and Arf-6 (55), have been shown to exhibit positive feedback. Vesicle budding and fusion processes (56) and protrusion and retraction of membrane structures such as filopodia and lamellipodia (57) as well as the formation of receptor signaling domains and protein condensates at the membrane (58) all represent dramatic changes in spatial confinement coupled with membrane signaling activity. All of these situations, and many others in cells, present viable opportunities for size-dependent reaction rates to be utilized in a regulatory mode.

## Materials and Methods

Procedures for protein purification, microfabrication, and all lipid bilayer experiments are included in *SI Appendix*.

### Stochastic Simulations.

The time evolution of all species in the reaction was simulated stochastically using the Gillespie algorithm. Within the reaction space, the membrane composition was approximated to be spatially homogeneous. The simulation was performed in MATLAB according to the kinetic scheme in Fig. 5*A*. We approximated the solution concentration of *E*_0_, $\rho_{E0}$, to be constant since in the experiment there is a large solution reservoir. Each molecular species is expressed as the exact number of molecules. The rate for each transition is calculated as follows:

$$E_{0}\;\to \;E_{1};r_{1}=k_{on}\cdot \rho_{E0}\cdot \sigma_{P}\cdot A,$$

$$E_{1}\;\to \;E_{0};r_{2}=\;k_{\mathrm{off}}\cdot \sigma_{E1}\cdot A,$$

$$E_{1}+S\;\to \;E_{1}S;r_{3}=\;k_{f}\cdot \sigma_{E1}\cdot \sigma_{S}\cdot A,$$

$$E_{1}S\to \;E_{1}+S;\;r_{4}=\;k_{r}\cdot \sigma_{E1S}\cdot A,$$

$$E_{1}S\to \;E_{1}+P;\;r_{5}=\;k_{\mathrm{cat}}\cdot \sigma_{E1S}\cdot A.$$

*A* is the area of the membrane in μm^2^, and the surface density of each membrane-associated species, $\sigma_{x}$, is expressed as discrete molecular copy number per unit area. We used the following rate parameters:

$$k_{on}\cdot \rho_{E0}=0.0001\;t^{-1}(\rho_{E0}\text{taken}\;\text{as}\;\text{constant}\;\text{for}\;\text{infinite}\;\text{solution}\;\text{reservoir}).,$$

$$k_{\mathrm{off}}=0.1\;t^{-1},$$

$$k_{f}=0.005\;{\mathrm{\mu m}}^{2}t^{-1},$$

$$k_{r}=1\;t^{-1},$$

$$k_{\mathrm{cat}}=50\;t^{-1}.$$

The kinetic parameters used are within similar ranges with reported kinetic rate constants for PTEN (26). All simulations begin with 26,600/μm^2^ substrate and 1,400/μm^2^ product (corresponding to 1.9% molar fraction of substrate and 0.1% molar fraction of product on the membrane) unless otherwise stated. This initial condition is used since in our simple model enzyme recruitment to the membrane is strictly through binding to product. For any enzyme to be recruited to the membrane, some product is required to “seed” the reaction in the simulation. This is to mimic the initial enzyme catalysis from the solution that starts the reaction, without introducing unnecessary complexity to the model. Simulations of 1 and 0.25 μm^2^ were used to mimic large- and small-scale membrane reactions, respectively. We note that a larger area difference amplifies any scale dependence in the simulations, though larger reactions require significantly more computation time. Statistics were collected from 1,000 simulations.

For the reaction case that has no positive feedback, the $E_{0}\;\to \;E_{1}$ rate is modified to be independent of $\sigma_{P}$:

$$E_{0}\;\to \;E_{1};r_{1}=k_{on}\cdot \rho_{E0}\cdot A.$$

The rate parameters used are as follows:

$$k_{on}\cdot \rho_{E0}=1.4\;t^{-1},$$

$$k_{\mathrm{off}}=0.1\;t^{-1},$$

$$k_{f}=0.005\;{\mathrm{\mu m}}^{2}t^{-1},$$

$$k_{r}=1\;t^{-1},$$

$$k_{\mathrm{cat}}=50\;t^{-1}.$$

For the reaction case that is fixed at steady state, the kinetic parameters used are as follows:

$$k_{on}\cdot \rho_{E0}=0.0001\;t^{-1},$$

$$k_{\mathrm{off}}=0.1\;t^{-1},$$

$$k_{f}=0.005\;{\mathrm{\mu m}}^{2}t^{-1},$$

$$k_{r}=1\;t^{-1},$$

$$k_{\mathrm{cat}}=50\;t^{-1}.$$

Simulations begin with 14,000/μm^2^ substrate and 14,000/μm^2^ product (corresponding to 1% molar fraction of substrate and 1% molar fraction of product on the membrane), and the densities are fixed. The formed product from the reaction is recorded separately to calculate the reaction velocity. The simulation was performed until the numbers reach a steady state. Then, the reaction was allowed to run for an extended time and was recorded.

For the reaction case with near-equilibrium enzyme binding, either the $k_{on}$ and $k_{\mathrm{off}}$ are changed to the following:

$$k_{on}\cdot \rho_{E0}=0.01\;t^{-1},$$

$$k_{\mathrm{off}}=10\;t^{-1},$$

or $k_{f}$ and $k_{\mathrm{cat}}$ are changed to the following:

$$k_{f}=0.0001\;{\mathrm{\mu m}}^{2}t^{-1},$$

$$k_{\mathrm{cat}}=1\;t^{-1}.$$

For the reaction case with near-equilibrium enzyme binding at low substrate density, simulations begin with 76/μm^2^ substrate and 4/μm^2^ product. Either the $k_{on}$ and $k_{\mathrm{off}}$ are changed to the following:

$$k_{on}\cdot \rho_{E0}=0.01\;t^{-1},$$

$$k_{\mathrm{off}}=10\;t^{-1}.$$

Or $k_{on}$, $k_{f}$, and $k_{\mathrm{cat}}$ are changed to the following:

$$k_{on}\cdot \rho_{E0}=0.02\;t^{-1},$$

$$k_{f}=0.0001\;\mu m^{2}t^{-1},$$

$$k_{\mathrm{cat}}=1\;t^{-1}.$$

For the reaction case with the incorporation of catalysis from solution by random collision of the enzyme with the membrane, we have included additional reactions:

$$E_{0}+S\;\to \;E_{0}S;r_{6}=k_{f2}\cdot \rho_{E0}\cdot \sigma_{s}\cdot A,$$

$$E_{0}S\to \;E_{0}+S;\;r_{7}=\;k_{r}\cdot \sigma_{E0S}\cdot A,$$

$$E_{0}S\to \;E_{0}+P;\;r_{8}=\;k_{\mathrm{cat}}\cdot \sigma_{E0S}\cdot A,$$

$$k_{f2}\cdot \rho_{E0}=0.003\;t^{-1}(\rho_{E0}\text{taken}\;\text{as}\;\text{constant}\;\text{for}\;\text{infinite}\;\text{solution}\;\text{reservoir}.).$$

Simulations begin with 28,000/μm^2^ substrate and 0/μm^2^ product.

### Deterministic Simulations.

Deterministic simulations were done by numerically solving coupled kinetic equations in MATLAB. Densities are evaluated as number of molecules per μm^2^, and the solution enzyme concentration, $\rho_{E0}$, is constant. The rate equations are as follows:

$$\frac{d\sigma_{E1}}{dt}=\;k_{on}\cdot \rho_{E0}\cdot \sigma_{P}\;-\;k_{\mathrm{off}}\cdot \sigma_{E1}-k_{f}\cdot \sigma_{E1}\cdot \sigma_{S}\;+\;k_{r}\cdot \sigma_{E1S}\;+\;k_{\mathrm{cat}}\cdot \sigma_{E1S},$$

$$\frac{d\sigma_{E1S}}{dt}=\;k_{f}\cdot \sigma_{E1}\cdot \sigma_{S}-\;k_{r}\cdot \sigma_{E1S}\;-\;k_{\mathrm{cat}}\cdot \sigma_{E1S},$$

$$\frac{d\sigma_{P}}{dt}=\;k_{\mathrm{cat}}\cdot \sigma_{E1S}\;-\;k_{on}\cdot \rho_{E0}\cdot \sigma_{P}\;+\;k_{\mathrm{off}}\cdot \sigma_{E1},$$

$$\frac{d\sigma_{S}}{dt}=\;-k_{f}\cdot \sigma_{E1}\cdot \sigma_{S}\;+\;k_{r}\cdot \sigma_{E1S}.$$

The rate constants are as follows:

$$k_{on}\cdot \rho_{E0}=0.0001\;t^{-1},$$

$$k_{\mathrm{off}}=0.1\;t^{-1},$$

$$k_{f}=0.005\;{\mathrm{\mu m}}^{2}t^{-1},$$

$$k_{r}=1\;t^{-1},$$

$$k_{\mathrm{cat}}=50\;t^{-1}.$$

Simulations begin with 26,600/μm^2^ substrate and 1,440/μm^2^ product (corresponding to 1.9% molar fraction of substrate and 0.1% molar fraction of product on the membrane).

### Analytical Model for Surface Adsorption.

In terms of a continuum description with deterministic chemical kinetic rate equations, the surface density of adsorbed molecules, σ, from an infinite solution reservoir follows the rate equation:

$$\frac{d\text{σ}}{dt}=\;k{\text{σ}}^{m},$$

where *k* is a constant, and *m* represents the order of positive feedback (e.g., *m* = 0 for no feedback, *m* = 1 for linear feedback, etc.). In this continuum description, the time for density doubling from $\sigma_{0}$ to $2\sigma_{0}$ ($\tau_{D}$) can be obtained by integrating the following rate equation:

$$\tau_{D}=\frac{1}{k}\overset{2\sigma_{0}}{\underset{\sigma_{0}}{\int }}\frac{1}{{\text{σ}}^{m}}d\text{σ}.$$

Taking a stochastic approach, the process of density doubling consists of a Markov chain of *n* molecular adsorption events (copy number *n* molecules goes to 2*n*), each with defined transition rates. We consider systems with different initial copy numbers of molecules, *n*, and correspondingly different areas, *A_n_*, at equivalent initial surface density to examine system size–specific effects. For a system starting with *n* molecules, the *i*^th^ transition has rate$\;k\sigma^{m}A_{n}$, where $\text{σ}=(n+i-1)/A_{n}$ is the momentary density of adsorbed molecules while waiting for the *i*^th^ transition event. The waiting time distribution at each step is given by $p_{i}\left(\tau_{i}\right)=\beta_{i}e^{-\beta_{i}\tau_{i}}$ where $\beta_{i}\equiv k\sigma^{m}A_{n}$ and $〈\tau_{i}〉=\int_{0}^{\infty }\tau_{i}p_{i}\left(\tau_{i}\right)d\tau_{i}=1/\beta_{i}$. The full probability distribution for $\tau_{D}$ resulting from successive convolution of the individual transition time probability distributions can be expressed in closed form as follows (59):

$$p\left(\tau_{D}\right)=\;\sum_{i=1}^{n}\frac{\beta_{1}\cdots \;\beta_{n}}{\prod_{\begin{array}{c} j=1\\ j\neq i \end{array}}^{n}(\beta_{j}-\;\beta_{i})}e^{-\beta_{i}\tau_{D}}.$$

Since the delay time probability distributions for each of the transitions are independent, the mean doubling time, equivalent to MFPT for this one-way process, can be calculated directly from the individual mean delay times, $〈\tau_{D}〉=\sum_{i=1}^{n}〈\tau_{i}〉$, without need for the full distribution.

## Supplementary Material

Supplementary File

Supplementary File

Supplementary File

Supplementary File

Supplementary File

Supplementary File

Supplementary File

Supplementary File

Supplementary File

Supplementary File

Supplementary File

Supplementary File

## Data Availability

All study data are included in the article and/or supporting information.
