# Supplementary material for: Stochasticity and positive feedback enable enzyme kinetics at the membrane to sense reaction size
Source: Proc Natl Acad Sci U S A. 2021 Nov 17;118(47):e2103626118. doi: 10.1073/pnas.2103626118 (PMC8617498; doi:10.1073/pnas.2103626118)
Supplement: Supplementary File [file pnas.2103626118.sapp.pdf]

PNAS
www.pnas.org

Supplementary Information for

Stochasticity and positive feedback enable enzyme kinetics at the membrane to sense reaction size

Albert A. Lee,[1,2] William Y. C. Huang,[1,4] Scott D. Hansen,[1,5] Neil H. Kim,[1] Steven Alvarez,[1,3] Jay T. Groves[1*]
[1]Department of Chemistry, University of California, Berkeley, CA, 94720, USA
[2]Department of Molecular and Cell Biology, University of California, Berkeley, CA, 94720, USA
[3]Department of Materials Science and Engineering, University of California, Berkeley, CA, 94720, USA
[4]Present address: Department of Chemical and Systems Biology, Stanford University, Stanford, CA, 94305, USA.
[5]Present address: Department of Chemistry and Biochemistry, University of Oregon, Eugene, OR, 97403, USA.
[*]Corresponding Author
Email: jtgroves@lbl.gov

This PDF file includes:

Materials and Methods

Supplementary Information text

Figures S1 to S14

Captions for Movie S1 to S11

Materials and Methods

Protein purification

PTEN

PTEN was cloned from PET30B-PTEN (PET30B-PTEN was a gift from Alonzo Ross (Addgene plasmid # 20741)(1). PTEN in-frame with a N-terminal his6-MBP-(Asn)10-TEV-GGGGG was transformed into BL21(DE3) expression cells, and these cells were grown overnight at 37ºC in TB medium supplemented with kanamycin (50μg/mL). The overnight culture was then diluted 1:100 (v/v) into fresh TB medium supplemented with kanamycin (50μg/mL), and was allowed to grow to OD600 of 0.6~0.8. Protein expression was induced with 0.1mM isopropyl β-D-1-thiogalactopyranoside (IPTG) and incubated at 18ºC 200rpm overnight. Bacterial cells were collected by centrifugation at 3000 rpm, resuspended in Lysis buffer (50 mM Na_2HPO_4 (pH 8.0), 300 mM NaCl, 0.4 mM BME, 1 mM PMSF, 100 μg/mL DNase) and homogenized using a microfluidizer. Cell debris was removed by centrifugation at 16,000 rpm at 4℃ for 60 minutes, and the supernatant was incubated with Ni-nitrilotriacetate resin (Thermo Scientific HisPur Ni-NTA Resin) at 4℃ for 1 hour. The resin was subsequently loaded into a gravity column, washed with Wash buffer (50 mM Na_2HPO_4 (pH 8.0), 300 mM NaCl, 1 mM BME, 20 mM imidazole), and eluted with Elution buffer (50 mM Na_2HPO_4 (pH 8.0), 300 mM NaCl, 1 mM BME, 500 mM imidazole). Peak fractions were pooled, combined with 200 μg/mL his6-TEV(S291V) protease, and dialyzed against 4 liters Dialysis buffer (20 mM Tris (pH 7.4), 200 mM NaCl, 1 mM BME) for 16-18 hours at 4℃. Precipitation was removed by centrifugation and 0.22 μm syringe filtration. The dialysate was then bound to a MonoQ anion exchange column (GE Healthcare) equilibrated in 20 mM Tris (pH 7.4), 100 mM NaCl, 1 mM BME. Proteins were resolved over a 10-100% linear gradient (0.1-1 M NaCl, 45 CV, 45 mL total, 1 mL/min flow rate). The elution fractions containing the recombinant protein were combined and concentrated in a 5 kDa MWCO Vivaspin 20 centrifuge tube (GE Healthcare). The concentration of protein was determined by measuring OD280 (ext. coefficient 45270.00 $M^{-1}cm^{-1}$) using nanodrop, and the protein solution was flash-frozen in liquid nitrogen and stored at -80ºC.

$PTEN_{\Delta PBD}$

PTEN(16-403) in-frame with an N-terminal his6-MBP-(Asn)10-TEV-GGGGG was transformed into BL21(DE3) expression cells, and these cells were grown overnight at 37ºC in TB medium supplemented with kanamycin (50μg/mL). The overnight culture was then diluted 1:100 (v/v) into fresh TB medium supplemented with kanamycin (50μg/mL), and was allowed to grow to OD600 of 0.6~0.8. Protein expression was induced with 0.1mM isopropyl β-D-1-thiogalactopyranoside (IPTG) and incubated at 18ºC 200rpm overnight. Bacterial cells were collected by centrifugation at 3000 rpm, resuspended in Lysis buffer (50 mM Na_2HPO_4 (pH 8.0), 300 mM NaCl, 0.4 mM BME, 1 mM PMSF, 100 μg/mL DNase) and homogenized using a microfluidizer. Cell debris was removed by centrifugation at 16,000 rpm at 4℃ for 60 minutes, and the supernatant was incubated with Ni-nitrilotriacetate resin (Thermo Scientific HisPur Ni-NTA Resin) at 4℃ for 1 hour. The resin was subsequently loaded into a gravity column, washed with Wash buffer (50 mM Na_2HPO_4 (pH 8.0), 300 mM NaCl, 1 mM BME, 20 mM imidazole), and eluted with Elution buffer (50 mM Na_2HPO_4 (pH 8.0), 300 mM NaCl, 1 mM BME, 500 mM imidazole). Peak fractions were pooled, combined with 200 μg/mL his6-TEV(S291V) protease, and dialyzed against 4 liters Dialysis buffer (20 mM Tris (pH 7.4), 200 mM NaCl, 1 mM BME) for 16-18 hours at 4℃. Precipitation was removed by centrifugation and 0.22 μm syringe filtration. The dialysate was then bound to a MonoQ anion exchange column (GE Healthcare) equilibrated in 20 mM Tris (pH 7.4), 100 mM NaCl, 1 mM BME. Proteins were resolved over a 10-100% linear gradient (0.1-1 M NaCl, 45 CV, 45 mL total, 1 mL/min flow rate). The elution fractions containing the recombinant protein were combined and concentrated in a 5 kDa MWCO Vivaspin 20 centrifuge tube (GE Healthcare), and subsequently pass through a 24 mL Superdex 200 10/300 GL (GE Healthcare) size exclusion column equilibrated in 20 mM Tris [pH 8.0], 200 mM NaCl, 10% glycerol, 1 mM TCEP. Peak fractions were pooled and concentrated. The concentration of protein was determined by measuring OD280 (ext. coefficient 45270.00 $M^{-1}cm^{-1}$) using nanodrop, and the protein solution was flash-frozen in liquid nitrogen and stored at -80ºC.

PTEN-DrrA
his6-MBP-(Asn)10-TEV-GGGGG-PTEN in frame with a C-terminal $(GGS)_3$GG-DrrA(544-647) was transformed into BL21(DE3) expression cells, and these cells were grown overnight at 37ºC in TB medium supplemented with kanamycin (50μg/mL). The overnight culture was then diluted 1:100 (v/v) into fresh TB medium supplemented with kanamycin (50μg/mL), and was allowed to grow to OD600 of 0.6~0.8. Protein expression was induced with 0.1mM isopropyl β-D-1-thiogalactopyranoside (IPTG) and incubated at 18ºC 200rpm overnight. Bacterial cells were collected by centrifugation at 3000 rpm, resuspended in Lysis buffer (50 mM Na_2HPO_4 (pH 8.0), 300 mM NaCl, 0.4 mM BME, 1 mM PMSF, 100 μg/mL DNase) and homogenized using a microfluidizer. Cell debris was removed by centrifugation at 16,000 rpm at 4℃ for 60 minutes, and the supernatant was incubated with Ni-nitrilotriacetate resin (Thermo Scientific HisPur Ni-NTA Resin) at 4℃ for 1 hour. The resin was subsequently loaded into a gravity column, washed with Wash buffer (50 mM Na_2HPO_4 (pH 8.0), 300 mM NaCl, 1 mM BME, 20 mM imidazole), and eluted with Elution buffer (50 mM Na_2HPO_4 (pH 8.0), 300 mM NaCl, 1 mM BME, 500 mM imidazole). Peak fractions were pooled, combined with 200 μg/mL his6-TEV(S291V) protease, and dialyzed against 4 liters Dialysis buffer (20 mM Tris (pH 7.4), 200 mM NaCl, 1 mM BME) for 16-18 hours at 4℃. Precipitation was removed by centrifugation and 0.22 μm syringe filtration. The dialysate was then bound to a MonoQ anion exchange column (GE Healthcare) equilibrated in 20 mM Tris (pH 7.4), 100 mM NaCl, 1 mM BME. Proteins were resolved over a 10-100% linear gradient (0.1-1 M NaCl, 45 CV, 45 mL total, 1 mL/min flow rate). The elution fractions containing the recombinant protein were combined and concentrated in a 5 kDa MWCO Vivaspin 20 centrifuge tube (GE Healthcare), and subsequently pass through a 24 mL Superdex 200 10/300 GL (GE Healthcare) size exclusion column equilibrated in 20 mM Tris [pH 8.0], 200 mM NaCl, 10% glycerol, 1 mM TCEP. Peak fractions were pooled and concentrated. The concentration of protein was determined by measuring OD280 (ext. coefficient 45270.00 $M^{-1}cm^{-1}$) using nanodrop, and the protein solution was flash-frozen in liquid nitrogen and stored at -80ºC.

$OCRL_{PD}$ & DrrA-$OCRL_{PD}$
$OCRL_{PD}$ (234-539aa) & DrrA-OCRLPD was purified as previously described(2).

Alexa488-$PLCδ_{PH}$ & Cy3-DrrA
PLCδ PH domain (11-140) and DrrA(544-647) were purified as previously described. Sortase mediated protein labeling was performed with either NHS-Alexa488 labeled LEPTGG peptide and NHS-Cy3 labeled LEPTGG as previously described(2).

$PIP5K_{KD}$
$PIP5K_{KD}$ (PIP5K1B 1-421aa) was purified as previously described(2).

Ras, SOS_{cat}, SOS_{HDPC} and p120GAP
H-Ras (1-181, C118S) (human H-Ras protein with residues 1-181 and a point mutation to serine at residue C118), SOS_{cat} (566-1049), SOS_{HDPC} (1-1049), and the GAP domain of p120GAP (714-1047), were expressed and purified based on the protocols described in previous work(3, 4).

Alexa647-RBD(K65E)
RBD (56-131; K65E) derived from the Raf-1 human gene was purified and labeled with Alexa647-maleimide using previously reported methods(5).

Preparation of liposome
Lipids used: 1,2-dioleoyl-sn-glycero-3-phosphocholine (DOPC), L-α-phosphatidylinositol-4-phosphate (Brain PI(4)P), L-α-phosphatidylinositol-4,5-bisphosphate (Brain PI(4,5)P_2), 1,2-dioleoyl-sn-glycero-3-phosphoethanolamine-N-[4-(pmaleimidomethyl)cyclohexanecarboxamide] (MCC-PE), 1,2-dioleoyl-sn-glycero-3-phospho-L-serine (DOPS), 1,2-dioleoyl-sn-glycero-3-phosphoethanolamine-N-(cap biotinyl) (Biotin-DOPE) were purchased from Avanti Polar Lipids. D-myo-Phosphatidylinositol 3,4,5-trisphosphate (PI(3,4,5)P_3), D-myo-Phosphatidylinositol 3,4-

bisphosphate (PI(3,4)P_2) were purchased from Echelon Biosciences Inc.. Texas Red 1,2-dihexadecanoylsnglycero-3-phosphoethanolamine (TR-DHPE) was purchased from Invitrogen.

Lipids were mixed in a glass round bottom flask cleaned by piranha etching at the desired molar fraction. The solution was then evaporated using a rotary evaporator for 10 min at 35°C or until dried to a thin film. Dried lipid films were further blow-dried with N_2 for at least 30 min. Lipids were resuspended in Milli-Q H_2O by shaking and gently pipetting to form a solution with a final concentration of 1 mM total lipids. For liposome assays, the lipid solution was first freeze-thawed three times in liquid nitrogen. To generate small unilamellar vesicles (SUV) with the desired size, the solution was extruded through a polycarbonate membrane (Avanti Polar Lipids) with the desired pore size 11 times. The size distribution was checked by dynamic light scattering. SUV for the formation of supported lipid bilayer was prepared by sonication for 100 sec (20 sec on, 30sec off for 5 times) in an ice-water bath.

Phosphatase assay
Phosphate Sensor (Thermo Fisher Scientific PV4406) was diluted to 2μM in TBS. 50 μL of liposomes were mixed with 25 μL of 2μM Phosphate Sensor in a 96-well plate. The background fluorescence of liposome mixed with phosphate sensor was taken as baseline. 25 μL of 40 nM PTEN was added into the 96-well plate to initiate the reaction. The fluorescent was detected in a microplate reader (BioTek) at excitation 485 (10) nm and emission 530 (10) nm with 30 seconds intervals, including 5 seconds of shaking before reading. A calibration curve for phosphate concentration was established using a phosphate standard from 10 pM to 100 nM and fitted to a binding curve. Initial velocity was obtained by fitting the initial linear region of the reaction trace. Initial velocity was plotted against SUV concentration, and fitted to Michaelis-Menten kinetics using $v = \frac{kcat[E][S]}{K_M+[S]}$ in Graphpad Prism. Apparent catalytic efficiency is obtained by calculating k_{cat}/K_M.

Microscope hardware and imaging acquisition
Fluorescence confocal microscopy was performed on Nikon Eclipse Ti inverted microscope with Yokogawa CSU-X spinning disk module. The light sources were diode lasers at 488, 561, and 640 nm (Coherent, Santa Clara, CA), and imaged by EMCCD (Andor Technology Ltd., UK) using a 100x Nikon objective (1.49 NA) oil immersion TIRF objective. TIRF imaging experiments were performed on an inverted Nikon Eclipse Ti microscope using either a 100x Nikon objective (1.49 NA) oil immersion TIRF objective or a 60x Apo TIRF oil immersion objective (1.45 NA). The light sources were either a 488 nm, 561 nm, or 637 nm diode laser (OBIS laser diode, Coherent, Santa Clara, CA) controlled with a custom built Solemere (Salt Lake City, Utah) laser driver with analog and digital modulation (0-5 volts). Images were acquired on an EMCCD camera (Andor Technology Ltd., UK). All microscope hardware was controlled using Micro-Manager v4.0(6). Samples were excited with 0.3~0.8 mW laser power at the objective. The exposure time is typically 100~200 ms. The imaging frame rate is typically 0.1 Hz.

Membrane coated beads experiments
200 uL of 10% slurry of Silica beads (Bangs Laboratories) were transferred to a glass vial. The beads were etched with piranha solution for 20 mins. The solution was diluted with water slowly on ice, then the beads were spun down by centrifugation in a swing bucket rotor at 1000 rcf for 5 min. The supernatant was removed by a glass pipette. The remaining beads were washed three times by water, then transferred to an Eppendorf tube. The beads were washed three times again in PBS. Finally, the beads were suspended in 200 uL of TBS. 20 uL of beads were mixed with 200 uL of PBS and 200 uL of 1 mM SUV solution (containing 96.5% DOPC, 2% PI(3,4,5)P_3, 1% Biotin-DOPE, 0.5% TR-DHPE) and incubated for 30 min with constant rotation. Then the beads were washed with PBS three times. Supported lipid bilayer coated beads were used for experiments immediately. 25x75 mm glass coverslips (Ibidi 10812) were etched with piranha solution for 5 minutes and rinsed with water extensively. The coverslip were dried with blowing nitrogen gas and stick to a flow chamber (Ibidi μ-Slide 80608). 100 uL of 1 mg/ml Biotin-BSA was introduced into the flow channel. After 30 min, the remaining Biotin-BSA was washed out with 1 mL of PBS. Blocking was performed with 0.05 mg/ml neutravidin was subsequently added. After 30 min, the remaining Biotin-BSA was

washed out with 1 mL of PBS. Membrane coated beads were added into the flow chamber and allowed to settle and bind to the neutravidin on the glass surface to achieve the desired density. Excessive beads were gently washed out using 1 mL PBS before imaging using a spinning disk confocal. Reaction was performed in a buffer containing 20 mM Tris (pH 7.4), 150 mM NaCl, 100 µg/mL beta casein, 5 mM BME, 2 mM UV-treated Trolox ((±)-6-Hydroxy-2,5,7,8-tetramethylchromane-2-carboxylic acid), 320 µg/mL glucose oxidase, 50 µg/mL catalase, and 20 mM glucose.

Microfabrication

25x75 mm glass coverslips (Ibidi 10812) were cleaned in acetone by sonication, then washed with MilliQ water extensively. The coverslips were dried with nitrogen gas then baked on 120℃ hot plate for 5 minutes. S1805 positive photoresist (Dow Chemical)) was spin-coated on the coverslips by spinning for 2 seconds at 500 rpm (acceleration 440 rpm/s) then for 30 seconds at 4111 rpm (acceleration 3900 rpm/s). The photoresist on the edge of the coverslips was removed by cotton swab soaked with acetone, then baked on 120℃ hot plate for 1 minute. Mask with the desired pattern was mounted on an OAI Series 200 Aligner. The photoresist coated coverslip was exposed for 0.6 sec with UV power around 30 mW/cm^2, then developed with MicroPosit MF-321 Liquid Developr (Dow Chemical) for 40 sec with mild shaking. The developed coverslips were rinsed with water and dried with nitrogen gas. ~9 nm thick chromium was subsequently deposited on the coverslips using an electron beam evaporator at 1x10^{-6} torr. The photoresist is lifted from chromium patterned glass substrates by bath sonication in MicroPosit Remover 1165 (Dow Chemical) for 10 minutes for 2 times then washed with abundant water.

Supported lipid bilayer experiments

Glass coverslips with chromium patterns were etched with piranha solution for 5 minutes and then rinsed with water extensively. The coverslip was rapidly dried with nitrogen gas and stick to a flow chamber (Ibidi µ-Slide 80608). SLBs were formed on a glass substrate by flowing around 150 µL of 0.25 mM SUVs diluted in PBS (pH 7.2) into the chamber and incubated for at least 30 min. After incubation, the chambers were washed with 1 mL of PBS and then blocked with 1 mg/mL β-casein (Thermo Fisher Scientific 37528) for 10 min. The chambers were then rinsed with 1 mL PBS buffer. PTEN reactions were performed in a buffer containing 20 mM Tris (pH 7.4), 150 mM NaCl, 100 µg/mL beta casein, 5 mM BME, 2 mM UV-treated Trolox, 320 µg/mL glucose oxidase, 50 µg/mL catalase, and 20 mM glucose, with the addition of 20 nM of either Alexa488-PLCδ$_{PH}$ or Cy3-DrrA to monitor the reaction. Fitting to Hill equation is performed in GraphPad Prism with the equation form: $Y = A \times \frac{X^H}{B^H + X^H}$.

For PIP5K and OCRL reactions, SUV containing either 96% DOPC, 2% PI(4,5)P$_2$, 2% PI(4)P, or 96% DOPC, 4% PI(4)P was used. Reactions were performed in a buffer containing 20 mM HEPES (pH 7.0), 150 mM NaCl, 1 mM ATP, 5 mM MgCl$_2$, 0.5 mM EGTA, 200 µg/mL beta casein, 20 mM BME, and 20 mM glucose, with the addition of 20 nM Alexa488-PLCδPH and 20 nM Cy3-DrrA to monitor the reaction.

For Ras reactions, SUV containing 96% DOPC, 2% PI(4,5)P$_2$, 2% MCC-DOPE were used to form supported lipid bilayer. H-Ras was incubated at 1 mg/mL for 2 hr 30 min in PBS buffer at room temperature. After washing with 1 mL PBS, 5 mM BME was then added to quench the reaction. After 10 min, the flow channel was washed with 1 mL of PBS, and buffer exchanged into 40 mM HEPES (pH 7.4), 100 mM NaCl, 5 mM MgCl$_2$, 100 µM GDP. GDP was washed away with 40 mM HEPES (pH 7.4), 100 mM NaCl, 5 mM MgCl$_2$, 10 mM BME immediately before reaction. Ras reactions were performed in a buffer containing 40 mM HEPES (pH 7.4), 100 mM NaCl, 5 mM MgCl$_2$, 10 mM BME, 100 µM GTP, 2 mM UV-treated Trolox, 320 µg/mL glucose oxidase, 50 µg/mL catalase, and 20 mM glucose, with the addition of 10 nM Alexa647-RBD(K65E) to monitor the reaction.

Statistics

The phosphatase experiment is repeated 3 times and averaged. Membrane coated bead experiment was repeated 3 times, the average was calculated form 6 beads. Micropatterned supported lipid bilayer experiments involves quantification from 100-500 corrals. The trend was confirmed with typically three repeats. All reported simulation condition was simulated 1000 times.

Supplementary Information Text

Hill equation fit

An estimate of feedback strength can be obtained by fitting the kinetic data to a Hill equation. For the PTEN reaction data in Fig. 2C, fitting to a general sigmoidal function (Hill equation) reveals the Hill slope is higher for the mean reaction trace in a free lipid bilayer compared to that for the mean reaction trace in 5 μm × 5 μm corrals (Fig. S11). This suggests that the reaction exhibits stronger feedback in the larger size reaction. When fitting the maximum reaction velocity versus PTEN concentration plot to a Hill equation, a higher Hill slope is observed in a free lipid bilayer compared to 5 μm × 5 μm corrals as well (Fig. S12).

Additional discussions on stochastic kinetic modeling

The stochastic simulation model (Fig. 5) is a minimal model that can still capture the size sensing behavior experimentally observed. It is not, however, a literal attempt to model the data and some differences are noted. Specifically, the simulations exhibit a noticeable spread in delay time before each trace starts to react, while in the experiment this is not observed. This results because, in the simple model, enzyme recruitment to the membrane is strictly through binding to its product at the membrane (see Material and Methods for further detail). In this case, the initial enzyme recruitment is limited by the low number of product molecules on the membrane, and will abruptly start the reaction once the first enzyme binds. In the experimental conditions, however, enzymes can slowly catalyze the reaction directly from the solution by random collisions, without being strongly anchored to the membrane. This provides a steady slow reaction rate before the first enzyme binds and smooth out the delay. This is demonstrated in simulations that incorporate an additional catalytic mechanism for the enzyme directly from the solution (see Material and Methods for further detail). These simulation results exhibit a delay time spread more reminiscent of the experimental data (Fig. S13). The artificially wide spread in start times in the simple model, however, averages to essentially the same mean when a large number of statistics are collected (1000 traces) and has no effect on the overall results. Moreover, the observed size dependency in mean reaction speed in the minimal model (Fig. 5) is not caused by the larger spread of reaction traces in small sizes (Fig. S14).

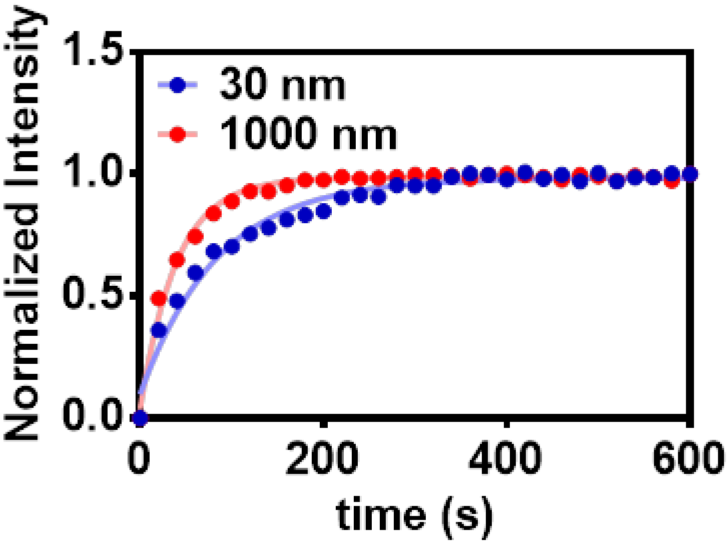

Fig. S1. Kinetic traces of PTEN catalyzed PI(3,4,5)P_3 reaction on 30 nm and 1000 nm liposomes. The reaction of 200 nM PTEN converting 10 µM of PI(3,4,5)P_3 to PI(4,5)P_2 monitored by phosphate release. Apparent rate constant k is obtained by fitting to 1-exp(-kx). 30 nm: k = 0.012; 1000 nm: k = 0.025

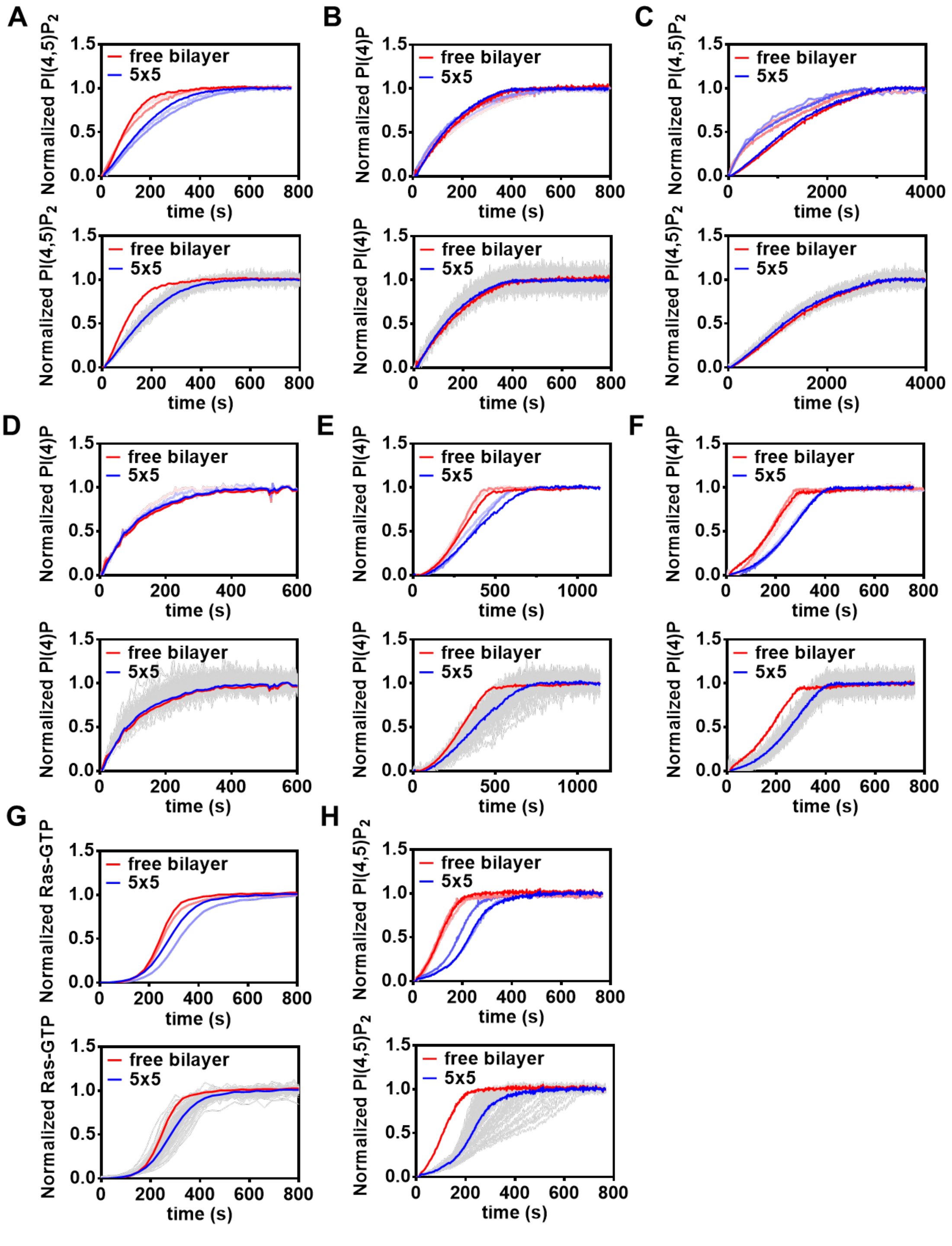

Fig. S2. Replicates and individual kinetic traces of corral assay. Top: Replicates of the corral assay. Bottom: Individual kinetic traces from a corral assay plotted with the mean. 100 individual traces in 5μm × 5μm corrals are plotted in grey. **(A)** $PI(3,4,5)P_3$ to $PI(4,5)P_2$ reaction catalyzed by 100 nM PTEN. **(B)** $PI(4,5)P_2$ to PI(4)P reaction catalyzed by 50 nM $OCRL_{PD}$. **(C)** $PI(3,4,5)P_3$ to $PI(4,5)P_2$ reaction catalyzed by 23 μM $PTEN_{\Delta PBD}$. **(D)** $PI(3,4)P_2$ to PI(4)P reaction catalyzed by 3 μM PTEN. **(E)** $PI(3,4)P_2$ to PI(4)P catalyzed by 170 nM PTEN-DrrA. **(F)** $PI(4,5)P_2$ to PI(4)P reaction catalyzed by 100 pM DrrA-$OCRL_{PD}$. **(G)** Ras-GDP to Ras-GTP reaction catalyzed by 20 nM SOS_{HDPC}. **(H)** PI(4)P to $PI(4,5)P_2$ catalyzed by 2 nM $PIP5K_{KD}$.

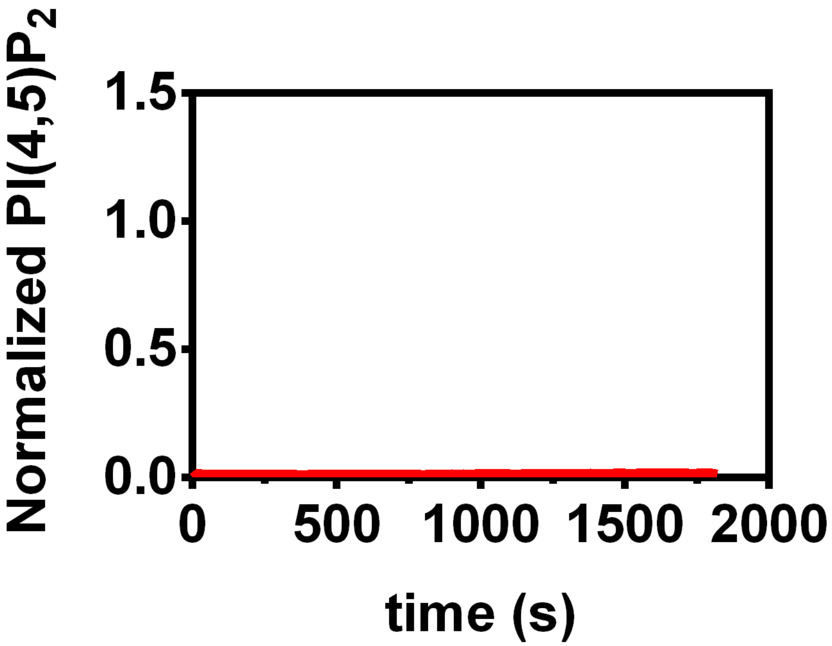

Fig S3. Reaction trace of $PTEN_{\Delta PBD}$ in the absence of PS. $PI(3,4,5)P_3$ to $PI(4,5)P_2$ reaction catalyzed by 20 μM $PTEN_{\Delta PBD}$ on free bilayer. Membrane composition is 98% DOPC. 2% $PI(3,4,5)P_3$.

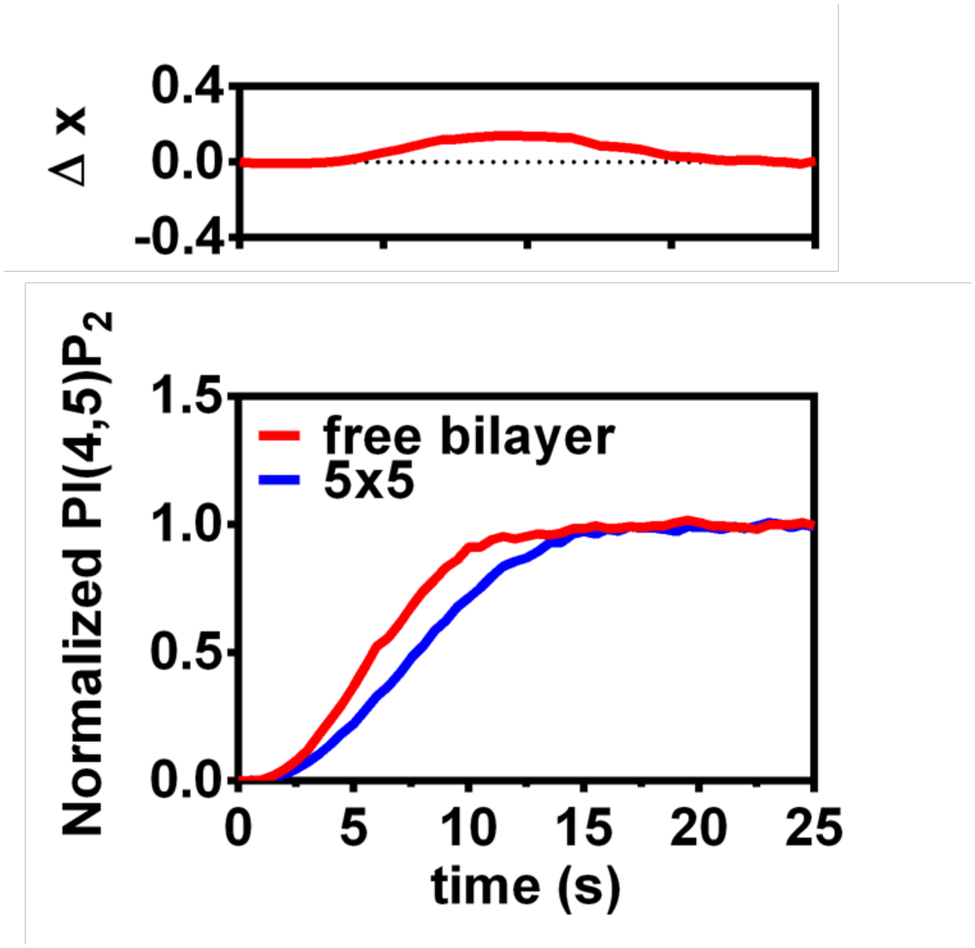

Fig S4. Reaction traces of PTEN in the presence of PS. Dephosphorylation reaction of $PI(3,4,5)P_3$ to $PI(4,5)P_2$ by 23 μM PTEN in 5μm x 5μm of membrane corrals and free bilayer. Membrane composition is 93% DOPC. 2% $PI(3,4,5)P_3$, and 5% PS.

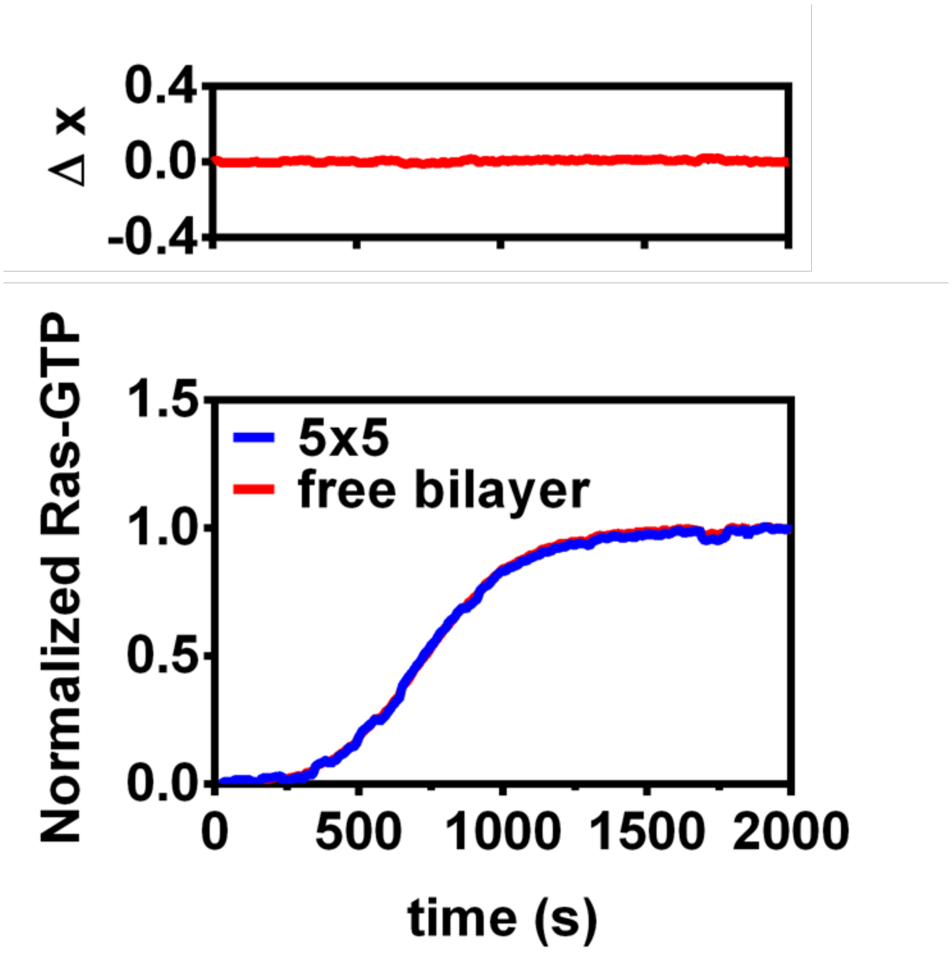

Fig S5. SOS_{cat} catalyzed Ras-GDP to Ras-GTP reaction. Nucleotide exchange reaction of Ras-GDP to Ras-GTP catalyzed by 2 nM SOS_{cat} in 5μm × 5μm membrane corrals and free bilayer.

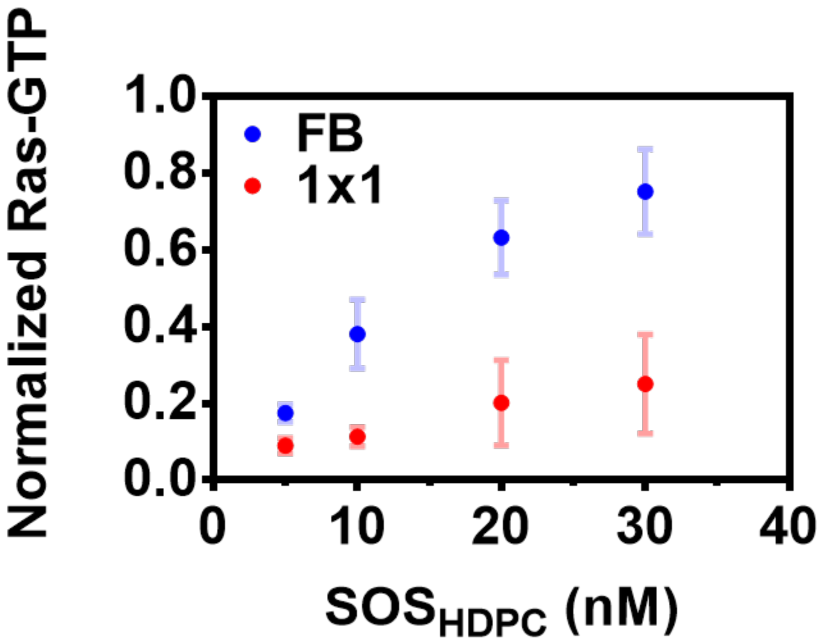

Fig S6. The steady-state level of Ras-GTP in SOS_{HDPC} and p120GAP competition reaction. SOS_{HDPC} competition with 200 nM p120GAP in1 μm × 1 μm membrane and free bilayer. Smaller reaction size leads to lower Ras-GTP steady state in the competition reaction.

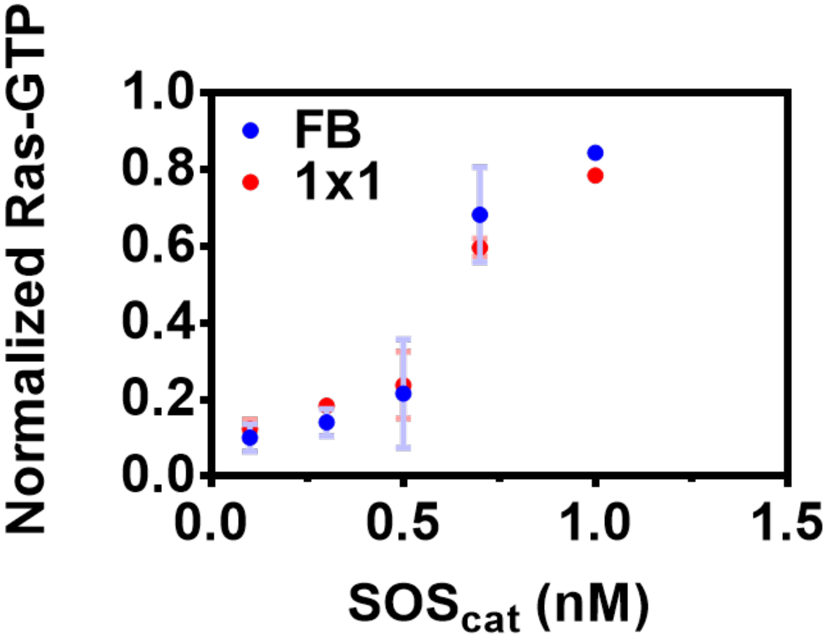

Fig S7. The steady-state level of Ras-GTP in SOS_{cat} and p120GAP competition reaction. SOS_{cat} competition with 200 nM p120GAP in 1 μm × 1 μm membrane and free bilayer. The Ras-GTP steady state of the competition reaction is independent of reaction size.

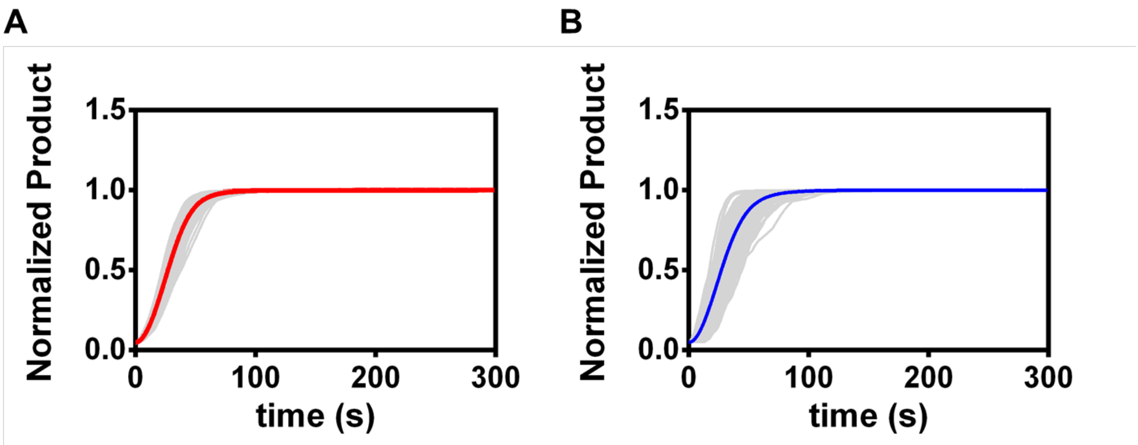

Fig S8. Individual kinetic traces from Fig. 5D (reaction with no positive feedback). (A) Kinetic traces from 1000 stochastic simulations in 1 μm^2 membrane plotted with their average. (B) Kinetic traces from 1000 stochastic simulations in 0.25 μm^2 membrane plotted with their average.

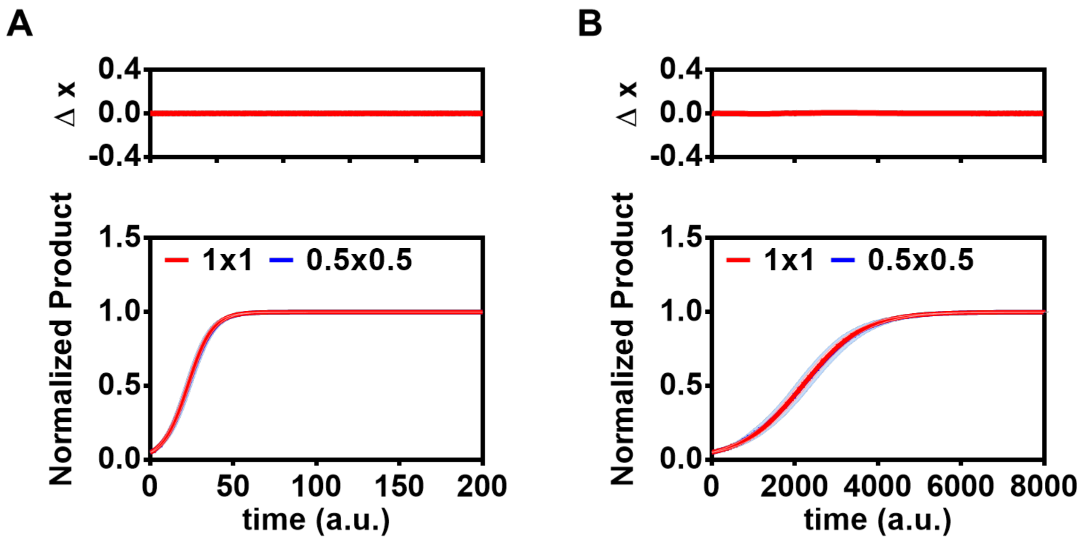

Fig S9. Stochastic simulation with near-equilibrium enzyme binding (non-processive enzyme catalysis) at high substrate density. (A) k_{on} and k_{off} were increased by 100 times, leading to a fast enzyme binding response to membrane composition change. **(B)** k_f and k_{off} were decreased by 50 times. Total substrate density is 28000/ μm^2.

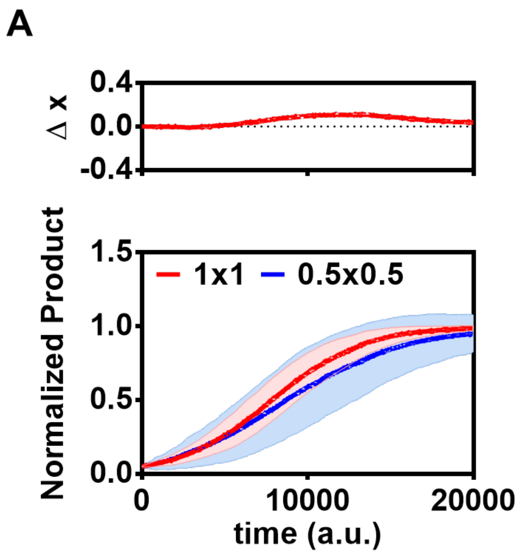

Fig S10. Stochastic simulation with near-equilibrium enzyme binding (non-processive enzyme catalysis) at low substrate density. (A) k_{on} and k_{off} were the same in Fig. S10A. **(B)** k_f and k_{off} were the same in Fig. S10B. Total substrate density is 80/ μm^2.

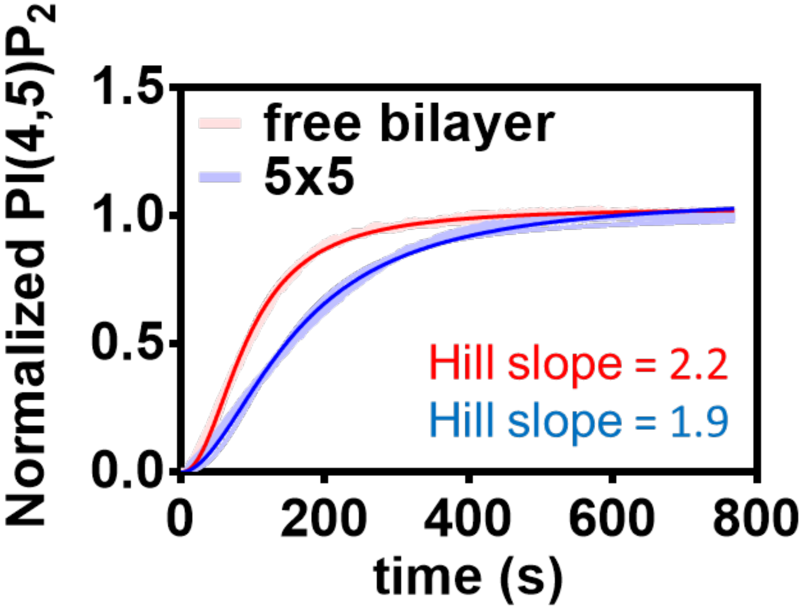

Fig S11. Analysis of the kinetic traces of PTEN reaction in Fig. 2C. The maximum velocity is 208/um^2/s in free lipid bilayer and 160/um^2/s in 5μm × 5μm corrals. Hill slope is 2.2 for the reaction in free lipid bilayer and 1.9 for the reaction in 5μm × 5μm corrals.

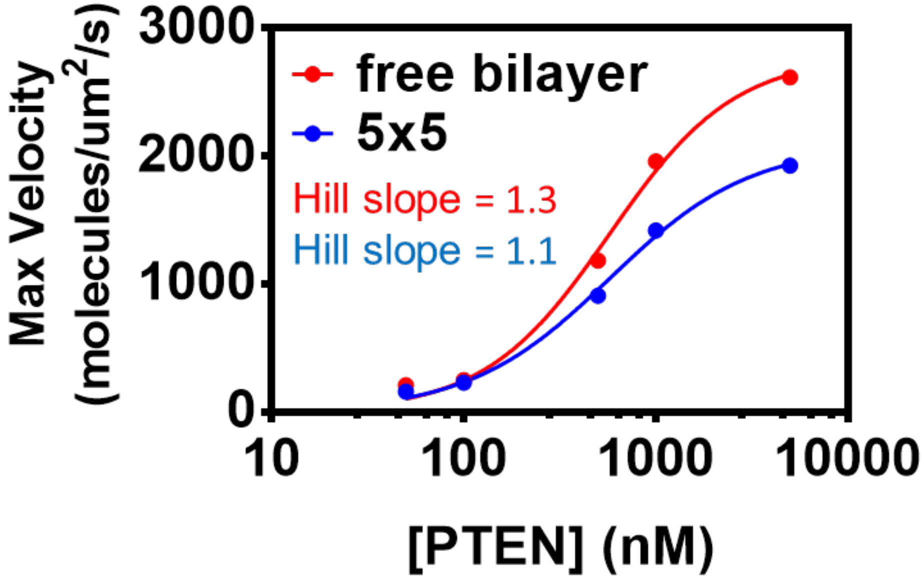

Fig S12. Maximum velocity versus PTEN concentration. Hill slope is 1.3 for the reaction in free lipid bilayer and 1.1 for the reaction in 5μm × 5μm corrals.

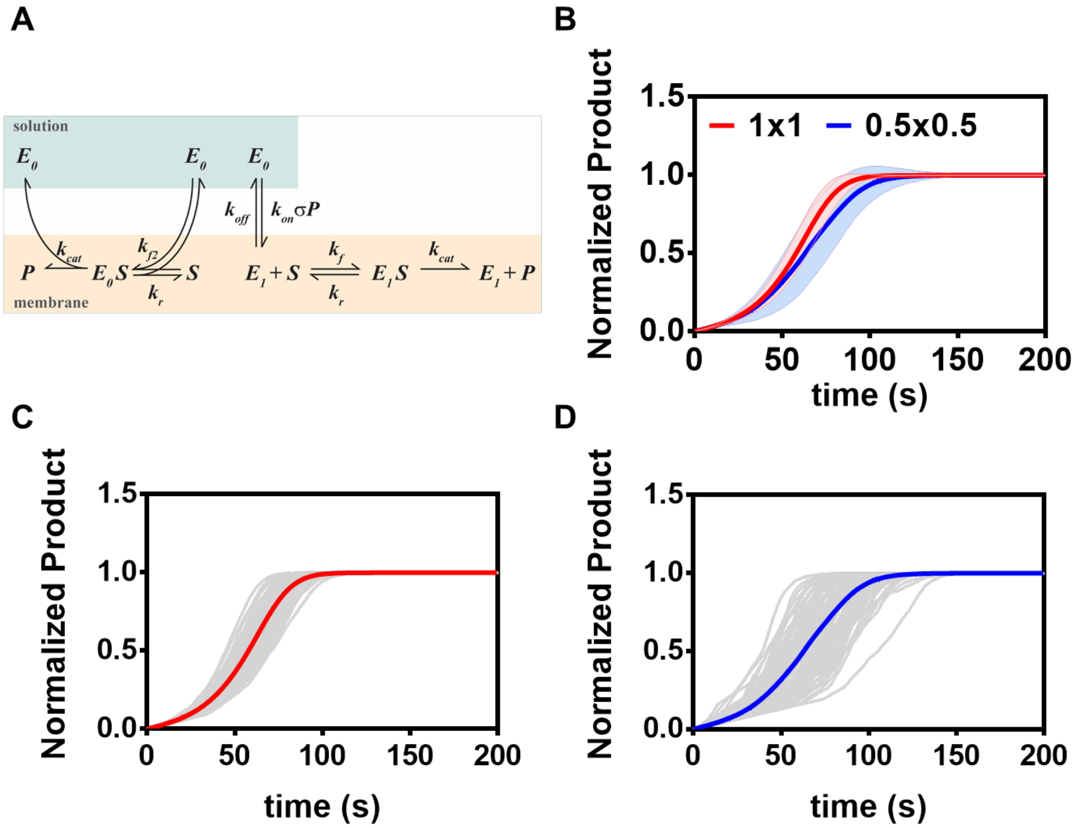

Fig S13. Stochastic simulation with the incorporation of additional catalysis mechanism from the enzyme, where the enzyme in the solution can randomly perform reaction without binding anchored to the product at the allosteric site. (A) Kinetic scheme for the stochastic kinetic modeling. In addition to the reactions described in Figure 5A, we have included the enzyme catalysis directly from the solution from random collision (see methods for details). (B) Average of kinetic traces from stochastic simulations using the reaction mechanism described in panel A in 1 μm × 1 μm (1 μm^2) membrane and 0.5 μm × 0.5 μm (0.25 μm^2) membrane. The shaded area shows the standard deviation. (C) Individual kinetic traces from 1000 stochastic simulations in 1 μm^2 membrane plotted with their average. (D) Individual kinetic traces from 1000 stochastic simulations in 0.25 μm^2 membrane plotted with their average.

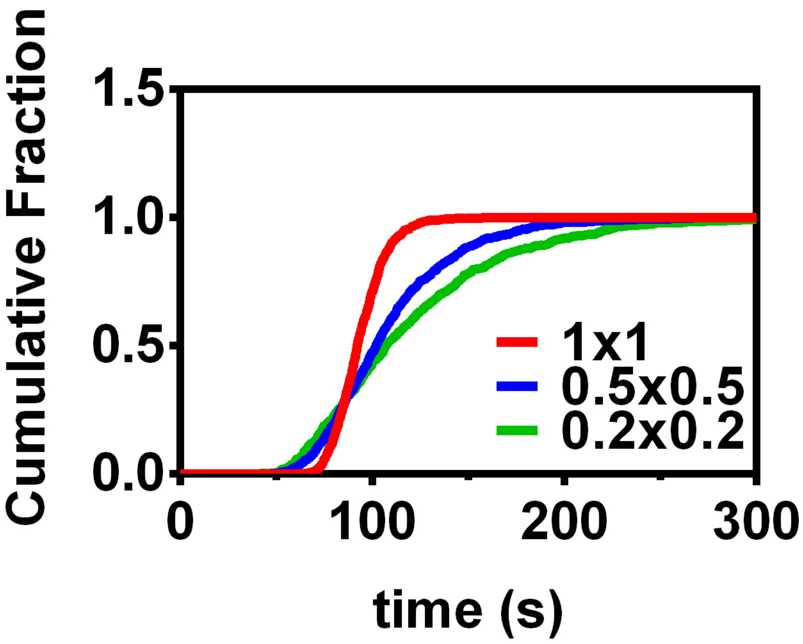

Fig S14. Cumulative distribution of time for simulated reactions in Fig. 5B to reach 95% reaction completeness. The median is at 0.5 cumulative fraction. Median: 92.1 a.u. for 1 μm^2; 101.9 a.u. for 0.25 μm^2; 107.2 a.u. for 0.16 μm^2.

Supplementary Movie Captions

Movie S1. Movie of 200 nM PTEN reaction on 6.89 µm beads and 2.34 µm beads. The fluorescence of Alexa488-PLCδ_{PH} is shown in green and the fluorescence of TR-DHPE is shown in red.

Movie S2. Movie of 100 nM PTEN reaction on supported lipid bilayer monitored by 20nM Alexa488-PLCδ_{PH}.

Movie S3. Movie of 50 nM $OCRL_{PD}$ reaction on supported lipid bilayer containing 4% PI(4,5)P2 monitored by 20nM Cy3-DrrA.

Movie S4. Movie of 23 µM $PTEN_{\Delta PBD}$ reaction on supported lipid bilayer monitored by 20nM Alexa488-PLCδ_{PH}.

Movie S5. Movie of 3 µM PTEN catalyzed PI(3,4)P_2 to PI(4)P reaction on supported lipid bilayer monitored by 20nM Cy3-DrrA.

Movie S6. Movie of 170 nM PTEN-DrrA catalyzed PI(3,4)P_2 to PI(4)P reaction on supported lipid bilayer monitored by 20nM Cy3-DrrA.

Movie S7. Movie of 100 pM DrrA-OCRL reaction on supported lipid bilayer monitored by 20nM Cy3-DrrA.

Movie S8. Movie of 2 nM $PIP5K_{KD}$ reaction on supported lipid bilayer monitored by 20nM Alexa488-PLCδ_{PH}.

Movie S9. Movie of 20 nM SOS_{HDPC} reaction on supported lipid bilayer monitored by 10nM Alexa647-RBD(K65E).

Movie S10. Movie of 2 nM SOS_{cat} reaction on supported lipid bilayer monitored by 10nM Alexa647-RBD(K65E).

Movie S11. Movie of competition reaction of 10 nM PIP5K and 700 nM OCRL on supported lipid bilayer monitored by 20 nM Alexa488-PLCδ_{PH} and 20 nM Cy3-DrrA. The fluorescence of Alexa488-PLCδ_{PH} is shown in yellow and the fluorescence of Cy3-DrrA is shown in blue.

References:

1. R. E. Redfern, *et al.*, PTEN phosphatase selectively binds phosphoinositides and undergoes structural changes. *Biochemistry* **47**, 2162–2171 (2008).

2. S. D. Hansen, *et al.*, Stochastic geometry sensing and polarization in a lipid kinase–phosphatase competitive reaction. *Proc. Natl. Acad. Sci. U. S. A.* **116**, 15013–15022 (2019).

3. J. Gureasko, *et al.*, Membrane-dependent signal integration by the Ras activator Son of sevenless. *Nat. Struct. Mol. Biol.* **15**, 452–461 (2008).

4. K. Scheffzek, *et al.*, The Ras-RasGAP Complex: Structural Basis for GTPase Activation and Its Loss in Oncogenic Ras Mutants. *Science (80-.).* **277**, 333–339 (1997).

5. W. Y. C. Huang, *et al.*, A molecular assembly phase transition and kinetic proofreading modulate Ras activation by SOS. *Science (80-.).* **363**, 1098–1103 (2019).

6. A. Edelstein, N. Amodaj, K. Hoover, R. Vale, N. Stuurman, Computer Control of Microscopes Using µManager. *Curr. Protoc. Mol. Biol.* **92**, 14.20.1-14.20.17 (2010).
